# Ketogenesis mitigates metabolic dysfunction–associated steatotic liver disease through mechanisms that extend beyond fat oxidation

**DOI:** 10.1172/JCI191021

**Published:** 2025-04-24

**Authors:** Eric D. Queathem, David B. Stagg, Alisa B. Nelson, Alec B. Chaves, Scott B. Crown, Kyle Fulghum, D. Andre d’Avignon, Justin R. Ryder, Patrick J. Bolan, Abdirahman Hayir, Jacob R. Gillingham, Shannon Jannatpour, Ferrol I. Rome, Ashley S. Williams, Deborah M. Muoio, Sayeed Ikramuddin, Curtis C. Hughey, Patrycja Puchalska, Peter A. Crawford

**Affiliations:** 1 Division of Molecular Medicine, Department of Medicine,; 2 Department of Biochemistry, Molecular Biology and Biophysics, and; 3 Department of Integrative Biology and Physiology, University of Minnesota Medical School, Minneapolis, Minnesota, USA.; 4 Duke Molecular Physiology Institute and Sarah W. Stedman Nutrition and Metabolism Center, Duke University Medical Center, Durham, North Carolina, USA.; 5 Department of Surgery, Lurie Children’s Hospital, Chicago, Illinois, USA.; 6 Department of Surgery, Northwestern University Feinberg School of Medicine, Chicago, Illinois, USA.; 7 Department of Radiology, University of Minnesota Medical School, Minneapolis, Minnesota, USA.; 8 Department of Surgery, University of Minnesota, Minneapolis, Minnesota, USA.; 9 Division of Endocrinology, Metabolism, and Nutrition, Department of Medicine, and; 10 Department of Pharmacology and Cancer Biology, Duke University Medical Center, Durham, North Carolina, USA.

**Keywords:** Hepatology, Metabolism, Fatty acid oxidation, Intermediary metabolism, Obesity

## Abstract

The progression of metabolic dysfunction–associated steatotic liver disease (MASLD) to metabolic dysfunction–associated steatohepatitis (MASH) involves alterations in both liver-autonomous and systemic metabolism that influence the liver’s balance of fat accretion and disposal. Here, we quantify the contributions of hepatic oxidative pathways to liver injury in MASLD-MASH. Using NMR spectroscopy, UHPLC-MS, and GC-MS, we performed stable isotope tracing and formal flux modeling to quantify hepatic oxidative fluxes in humans across the spectrum of MASLD-MASH, and in mouse models of impaired ketogenesis. In humans with MASH, liver injury correlated positively with ketogenesis and total fat oxidation, but not with turnover of the tricarboxylic acid cycle. Loss-of-function mouse models demonstrated that disruption of mitochondrial HMG-CoA synthase (HMGCS2), the rate-limiting step of ketogenesis, impairs overall hepatic fat oxidation and induces an MASLD-MASH–like phenotype. Disruption of mitochondrial β-hydroxybutyrate dehydrogenase (BDH1), the terminal step of ketogenesis, also impaired fat oxidation, but surprisingly did not exacerbate steatotic liver injury. Taken together, these findings suggest that quantifiable variations in overall hepatic fat oxidation may not be a primary determinant of MASLD-to-MASH progression, but rather that maintenance of ketogenesis could serve a protective role through additional mechanisms that extend beyond overall rates of fat oxidation.

## Abstract 2

These findings suggest that quantifiable variations in overall hepatic fat oxidation may not be a primary determinant of MASLD-to-MASH progression.

## Introduction

The global prevalence of metabolic dysfunction–associated steatotic liver disease (MASLD) is surging and is associated with mortality and multiple comorbidities (1, 2). The natural history of MASLD is tightly linked to obesity and insulin resistance, and involves both liver-autonomous and systemic metabolic abnormalities that drive the ectopic storage of triacylglycerol (TAG) species in hepatocytes (i.e., hepatic steatosis), which is strongly associated with cellular injury, inflammation, and the development of metabolic dysfunction–associated steatohepatitis (MASH), a progressive fibrotic liver disease that markedly increases the risks of cirrhosis and hepatocellular carcinoma (3–5). While the severity of hepatic steatosis correlates with the extent of liver injury, the pathogenesis of MASLD-MASH progresses through multiple parallel and mutually amplifying pathways. For any degree of steatosis, the development of MASH is marked by the appearance of oxidative and inflammatory stress, coupled to hepatocellular injury (i.e., hepatocyte ballooning), which collectively is quantified using the NAFLD (nonalcoholic fatty liver disease, the prior term for MASLD) activity score (NAS), a histological scoring system ranging 0 to 8, which encompasses steatosis (scores of 0–3), inflammation (scores of 0–3), and hepatocyte ballooning (scores of 0–2). MASH is defined by a NAS of ≥4, and typically coincides with fibrosis, though fibrosis is not included in calculation of NAS (6).

Currently there is only one FDA-approved drug available for the treatment of MASLD-MASH, while several others have failed owing to poor efficacy and/or toxicity, illustrating a need to understand the drivers of hepatic fat accumulation and its mechanistic link to the development of injury and advanced liver disease (7, 8). Though incretin mimetics and sodium-glucose cotransporter 2 (SGLT2) inhibitors show promise for MASLD, an unmet need remains for approaches that (a) are liver specific, indicated for patients without diabetes or obesity; (b) limit lifelong high-cost pharmacotherapy; and/or (c) limit toxicities (9, 10). Prior studies have correlated liver TAGs in obesity and/or MASLD to a rise in hepatic de novo lipogenesis (11–16), a diminution in polyunsaturated fatty acids (17–21), and an acceleration in hepatic oxidative fluxes (22–25). Other studies have shown no changes in hepatic fat oxidation (26–29), or have reported impairments in oxidative metabolism (30–34). Therefore, the lack of consensus regarding alterations in hepatic oxidative fluxes throughout the natural history of MASLD-MASH illustrates one of the key obstacles in developing effective therapeutics.

Oxidative metabolism in the liver yields reducing equivalents (REs) in the forms of reduced nicotinamide adenine dinucleotide (NADH) and flavin adenine dinucleotide (FADH_2_), which are primarily sourced from hepatic fat oxidation via (a) β-oxidation, which generates acetyl-CoA, and (b) terminal oxidation of acetyl-CoA in the tricarboxylic acid (TCA) cycle (35). RE production is also directly coupled to ATP production, which fuels phosphoenolpyruvate-derived (PEP-derived) gluconeogenesis (GNG) via the non-oxidative entry (anaplerosis) and exit (cataplerosis) of intermediates through the TCA cycle (36). In both human and rodent models of MASLD, hepatic TCA cycle turnover and PEP-derived GNG are increased relative to controls (25, 36, 37). Very low-density lipoprotein (VLDL) secretion rises with fasting and early in the natural history of steatotic liver disease (38, 39). However, VLDL secretion rates fail to compensate for TAG accumulation with progressive MASLD, and VLDL secretion is intrinsically impaired in humans harboring the PNPLA3 I148M mutation (40–42). While the stimulation of fat disposal pathways may offload excess TAGs, the rise in lipid disposal is insufficient to compensate for the rate of lipid appearance, leading to ectopic fat accumulation and predisposition to injury. In addition to VLDL secretion and terminal oxidation in the TCA cycle, ketogenesis, which produces d-β-hydroxybutyrate (d-βOHB) and acetoacetate (AcAc), is a major conduit supporting hepatic fat disposal. Congenital deficiency of the fate-committing enzyme of ketogenesis, 3-hydroxymethylglutaryl-CoA (HMG-CoA) synthase 2 (HMGCS2), is linked to hepatomegaly and steatosis (43, 44). Moreover, programmed ketogenic insufficiency in mice is associated with more aggressive MASLD progression (45–49). However, the relative rate of ketogenesis in MASLD, compared with controls, has varied among studies (22, 27, 34, 37, 50). As such, whether alterations in ketogenesis in human MASLD-MASH play driver, bystander, or compensating roles is debated, as are the mechanisms that link ketogenesis to oxidative metabolism and liver injury. To date, in vivo hepatic oxidative fluxes have only been quantified in humans with uncomplicated MASLD, but have not been measured in humans with histologically confirmed MASH. To understand the drivers and/or predictors of MASLD-MASH development, progression, and resolution, we quantified hepatic oxidative fluxes in histologically confirmed MASH patients using nuclear magnetic resonance (NMR) spectroscopy, ultra-high-performance liquid chromatography coupled to mass spectrometry (UHPLC-MS), and ^2^H/^13^C stable isotope tracing. Ketogenesis-insufficient mouse models were used to determine the relationships among ketogenesis, the rate of overall fat oxidation, and liver injury. Together, our observations are consistent with the notion that while ketogenesis and fat oxidation are key inputs into the liver’s balance of lipid accretion and disposal, the driver role ketogenesis plays in the rate of hepatic fat oxidation may not be the only determinant of MASLD-to-MASH progression. Therefore, maintenance of hepatic ketogenesis could serve additional protective roles through mechanisms extending beyond its contribution to the rate of hepatic fat oxidation.

## Results

### Hepatic ketogenesis correlates with NAS in humans.

**Figure 1:**
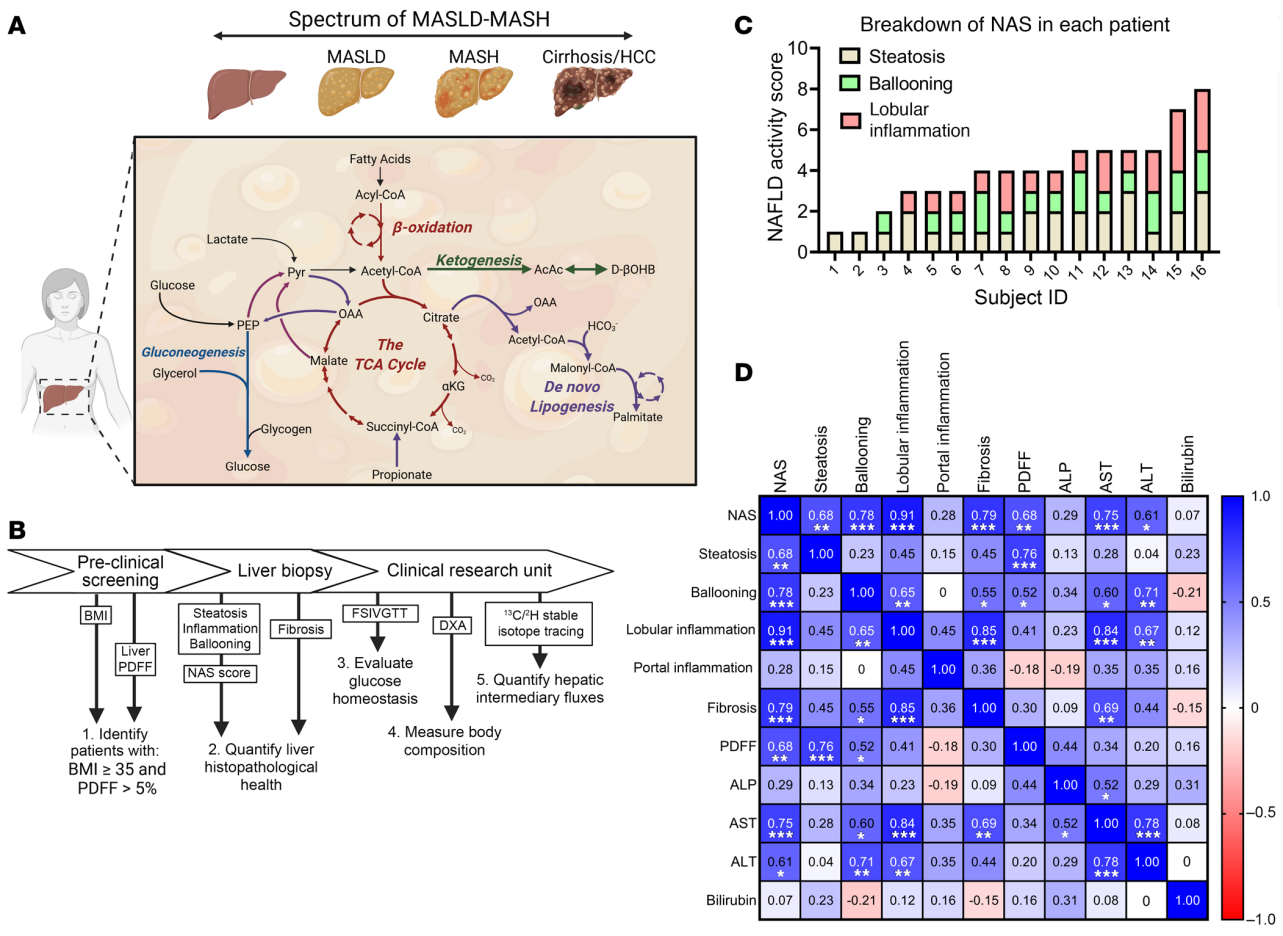
Metabolic characteristics of MASLD-MASH. (**A**) The initial stages of metabolic dysfunction–associated steatotic liver disease (MASLD) begin with hepatic steatosis, linked to accelerations in: de novo lipogenesis (DNL), turnover of the tricarboxylic acid (TCA) cycle, and phosphoenolpyruvate-derived (PEP-derived) gluconeogenesis (GNG). Metabolic shifts during the progression of MASLD to metabolic dysfunction–associated steatohepatitis (MASH) remain poorly understood. HCC, hepatocellular carcinoma. (**B**) Study design. Patients with BMI ≥ 35 and liver proton density fat fraction (PDFF) greater than 5% underwent a liver biopsy, FSIVGTT, and DXA imaging. After an overnight fast, 8 hepatic intermediary metabolic fluxes were quantified using ^2^H/^13^C stable isotope tracing. (**C**) Distributions of the NAFLD activity scores (NASs) in all 16 participants (15 female, 1 male). (**D**) Correlation matrix of NAS with histological scores for steatosis, ballooning, inflammation, fibrosis, PDFF, liver enzymes, and bilirubin. Pearson’s correlation coefficients (*r*) are shown in heatmap format with the magnitude of the correlation given by the right-hand legend and displayed in each square. Correlations were accepted as significant if *P* < 0.05. *P < 0.05, **P < 0.01, ***P < 0.001. ALP, alkaline phosphatase; AST, aspartate aminotransferase; ALT, alanine transaminase.

During the progression of MASLD, reports of hepatic oxidative fluxes have been varied, with studies reporting accelerations (22–25), no changes (26–29), or impairments (30–34) in oxidative metabolism. However, oxidative fluxes have never been quantified in vivo in humans with histologically confirmed MASH (Figure 1A). To study hepatic metabolism in humans with MASH, participants were recruited from 2 clinical trials, NCT03997422 and NCT03587831 (Supplemental Table 1; supplemental material available online with this article; https://doi.org/10.1172/JCI191021DS1). Recruited participants remained weight stable during metabolic assessments. Participants with BMI ≥ 35 were screened with magnetic resonance imaging to identify patients with a liver proton density fat fraction (PDFF) greater than 5%, indicative of hepatic steatosis, and a total of 16 participants (15 female and 1 male) were recruited to the study outlined in Figure 1B. After liver biopsy, glucose homeostasis and body composition were quantified using a frequently sampled intravenous glucose tolerance test (FSIVGTT) and dual-energy x-ray absorptiometry (DXA), respectively. The cohort’s characteristics are summarized in Table 1. During a 20-hour fast, oral ^2^H_2_O and [U-^13^C_3_]propionate tracers were delivered to enrich plasma glucose across hydrogen and carbon atoms, whose positional isotopomeric labeling distribution supports modeling of relative reaction velocities (V) (i.e., flux) through hepatic intermediary metabolic pathways. Simultaneously, the absolute rates of endogenous glucose production (EGP) (V_EGP_) and ketogenesis (V_RaβOHB_) were measured at metabolic and isotopic steady state after a 2-hour infusion of [3,4-^13^C_2_]glucose and d-[U-^13^C_4_]βOHB, respectively, thereby allowing the absolute rates of hepatic intermediary metabolic pathways to be quantified (Supplemental Figure 1) (35, 51–53). During the screening phase, and prior to flux assessments, a liver biopsy was collected and used to histologically grade liver health, which was collectively summarized by the NAS. NAS scores ranged from 1 to 8, all patients exhibited histological signs of steatosis, and 14 of 16 participants displayed signs of hepatocyte cell injury (i.e., ballooning) and/or lobular inflammation (Figure 1C). Ten of the 16 participants exhibited histopathological fibrosis. As expected, NAS strongly correlated with PDFF (*r* = 0.68, *P* < 0.01) and liver fibrosis (*r* = 0.79, *P* < 0.001) (Figure 1D), but did not correlate with BMI (Supplemental Figure 2A). NAS correlated with the acute insulin response to glucose (AIRg) (*r* = 0.68, *P* = 0.005), suggestive of a correlation between insulin resistance and NAS (Supplemental Figure 2B); however, NAS did not correlate with the homeostatic model assessment for insulin resistance (Supplemental Figure 2C).

**Figure 2:**
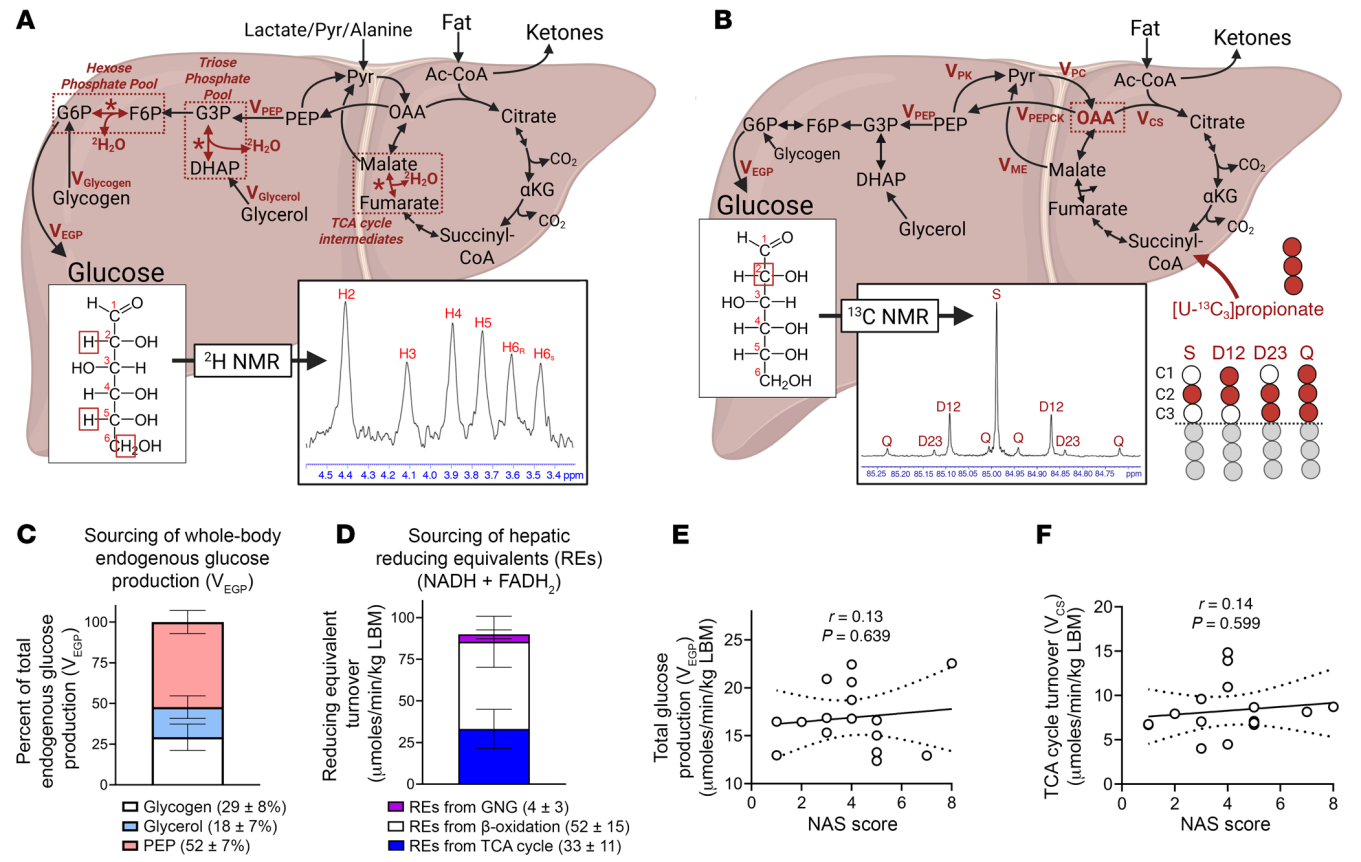
Liver injury does not correlate with endogenous glucose production (EGP) or TCA cycle turnover. Fluxes through hepatic intermediary metabolic pathways were quantified in humans after oral administration of heavy water (^2^H_2_O) and [U-^13^C_3_]propionate. [3,4-^13^C_2_]Glucose and d-[U-^13^C_4_]βOHB were intravenously infused, allowing whole-body glucose (V_EGP_) and βOHB turnover (V_RaβOHB_) to be quantified at metabolic and isotopic steady state. (**A**) Fractional sourcing of glucose can be quantified from the ^2^H enrichment pattern of plasma glucose using ^2^H NMR, which, by multiplying by V_EGP_, allows absolute reaction velocities (V) (i.e., flux) for hepatic glucose sourcing pathways to be quantified. (**B**) Administration of [U-^13^C_3_]propionate ^13^C-enriches TCA cycle intermediates, which sources phosphoenolpyruvate (PEP). Using ^13^C NMR, and the multiplet arising from the C2 resonance of plasma glucose, the resulting metabolic network models oxidative and anaplerotic nodes of the TCA cycle in parallel to glucose production. By normalizing of fluxes to V_PEP_, the absolute reaction velocities of the TCA cycle, anaplerosis/cataplerosis, and pyruvate cycling can be quantified. (**C**) Average percentage of V_EGP_ derived from glycogen, glycerol, and PEP, highlighting that TCA cycle–sourced PEP is the major contributor to V_EGP_ in the fasted state in humans. (**D**) Average reducing equivalents (REs) derived from GNG, β-oxidation, and the TCA cycle. (**E** and **F**) The correlations of NAS with V_EGP_ (**E**) and TCA cycle turnover (V_CS_) (**F**). Data are either expressed as mean ± SD or shown as correlations. Pearson’s correlation coefficients (*r*) are shown on each group along with a line of best fit and 95% confidence intervals calculated using linear regression. Correlations were accepted as significant if *P* < 0.05. *P* values are shown on each graph.

We then examined the metabolic flux modeling data acquired in this cohort (Figure 2, A and B). Across participants, 29% ± 8% of V_EGP_ arose from glycogenolysis (V_glycogen_), 18% ± 7% from glycerol (V_glycerol_), and 52% ± 7% from PEP (V_PEP_) (Figure 2C). Next, by quantification of rates of (a) TCA cycle flux, V_CS_ (where CS stands for citrate synthase); (b) total ketone body (TKB) production, V_RaTKB_; and (c) GNG sourcing pathways, V_glycerol_ and V_PEP_, both hepatic fat oxidation and RE production were calculated. The majority of REs produced in the fasted liver originated from β-oxidation, accounting for 52 ± 15 μmol REs/min/kg lean body mass (LBM), while REs derived from the TCA cycle accounted for 33 ± 11 μmol REs/min/kg LBM, and GNG accounted for 4 ± 3 μmol REs/min/kg LBM (Figure 2D). To determine relationships among fluxes, we performed regression analyses and obtained Pearson’s correlation coefficients (Supplemental Figure 3A). As expected, V_EGP_ correlated with V_glycogen_, V_PEP_, and total anaplerosis, V_PEPCK_ (where PEPCK stands for PEP carboxykinase). Moreover, expected correlations were observed between V_PEPCK_ and pyruvate cycling, V_PK+ME_ (where PK stands for pyruvate kinase and ME stands for malic enzyme) (*r* = 0.99, *P* < 0.0001); between V_CS_ and V_PK+ME_ (*r* = 0.60, *P* = 0.014); and between V_CS_ and V_PEPCK_ (*r* = 0.65, *P* = 0.006) (Supplemental Figure 3B). Additional correlations were observed between V_EGP_ and V_PK+ME_ (*r* = 0.63, *P* = 0.009), V_EGP_ and V_PEPCK_ (*r* = 0.70, *P* = 0.003), and V_EGP_ and V_CS_ (*r* = 0.57, *P* = 0.020) (Supplemental Figure 3C), as well as V_PEP_ and V_PK+ME_ (*r* = 0.68, *P* = 0.004), V_PEP_ and V_PEPCK_ (*r* = 0.79, *P* < 0.001), and V_PEP_ and V_CS_ (*r* = 0.68, *P* = 0.004) (Supplemental Figure 3D). Inverse correlations were observed between the percentage of V_EGP_ attributable to glycerol (V_glycerol_) and V_PK+ME_, V_PEPCK_, and V_CS_ (Supplemental Figure 3E). No correlations were observed between the ketogenic flux V_RaβOHB_ and any individual flux (Supplemental Figure 3A). As expected, total hepatic ketogenesis (V_RaTKB_) correlated with total hepatic fat oxidation (*r* = 0.96, *P* < 0.001), suggesting a driver role of ketogenesis in determining overall rates of hepatic fat oxidation (Supplemental Figure 3F). Interestingly, total fat oxidation was not correlated with V_CS_ (*r* = 0.22, *P* = 0.413) (Supplemental Figure 3G). Taken together, these data reflect internal consistency in the modeling data, demonstrating expected relationships between TCA cycle flux, GNG, ketogenesis, and total fat oxidation.

**Figure 3:**
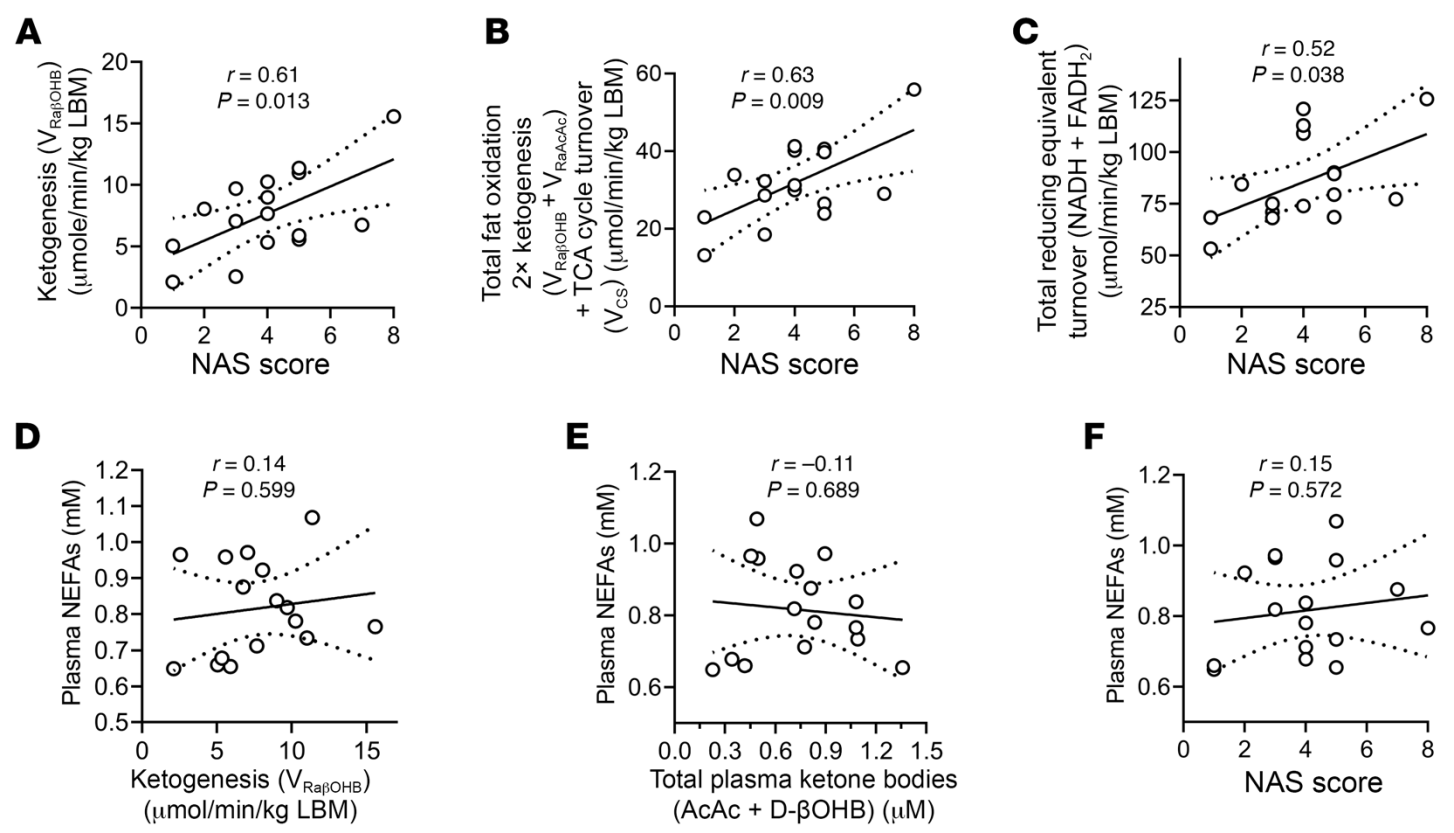
Liver injury correlates with endogenous ketogenesis and hepatic fat oxidation. (**A**–**C**) The correlation of NAS with endogenous ketogenesis (V_RaβOHB_) (**A**), total fat oxidation (**B**), and total RE turnover (**C**). (**D**–**F**) Correlation of serum non-esterified fatty acid (NEFA) concentrations with endogenous ketogenesis (V_RaβOHB_) (**D**), total plasma ketone bodies (AcAc + βOHB) (**E**), and NAS (**F**). Pearson’s correlation coefficients (*r*) are given on each graph along with a line of best fit calculated using linear regression. Correlations were accepted as significant if *P* < 0.05. *P* values are shown on each graph.

Given the validity of the flux modeling data, we next sought to determine the relationships among these measured fluxes and MASLD-MASH progression. Surprisingly, despite the known relationship among insulin resistance, V_EGP_, and NAS, a correlation between V_EGP_ and NAS was not detected (*r* = 0.13, *P* = 0.639) (Figure 2E). Moreover, V_CS_, V_glycogen_, V_glycerol_, V_PEP_, V_PK+ME_, and V_PEPCK_ all showed no correlation with NAS (Figure 2F and Supplemental Figure 4, A and B). However, surrogates for ketogenesis correlated directly with NAS (V_RaβOHB_ [*r* = 0.61, *P* = 0.013], Figure 3A; V_RaAcAc_ [*r* = 0.58, *P* = 0.018], V_RaTKB_ [*r* = 0.61, *P* = 0.013], and serum total [^12^C]ketone bodies [*r* = 0.53, *P* = 0.037], Supplemental Figure 4C). Consistent with the correlation between ketogenesis and total fat oxidation (Supplemental Figure 3F), both total fat oxidation (*r* = 0.63, *P* = 0.009) and total RE production (*r* = 0.52, *P* = 0.038) also correlated directly with NAS (Figure 3, B and C). The mitochondrial redox ratio of βOHB to AcAc did not correlate with NAS (Supplemental Figure 4D). V_RaβOHB_ did not correlate with liver PDFF (*r* = 0.38, *P* = 0.143), but trended toward a positive correlation with AIRg (*r* = 0.46, *P* = 0.081) (Supplemental Figure 5, A and B). V_RaβOHB_ also positively correlated with circulating transaminase levels (aspartate aminotransferase, *r* = 0.51, *P* = 0.041; alanine transaminase, *r* = 0.52, *P* = 0.037) (Supplemental Figure 5C). Despite the observed correlations with ketogenesis, similar correlations were not observed for circulating non-esterified fatty acids (NEFAs), a primary substrate for ketogenesis. Circulating NEFAs did not correlate with ketogenesis (V_RaβOHB_) or with total plasma ketone bodies (Figure 3, D and E), suggesting that variations in ketogenesis were not driven by variations in the availability of NEFAs in these participants under these conditions. Circulating NEFAs also did not correlate with NAS (*r* = 0.15, *P* = 0.572) (Figure 3F). Moreover, NEFAs also did not correlate with whole-body fat mass or percentage android fat, but did correlate with percentage gynoid fat (*r* = 0.50, *P* = 0.050) and inversely correlated with the android/gynoid ratio (*r* = –0.55, *P* = 0.028) (Supplemental Figure 5D).

Though not included in NAS, fibrosis is also a strong indicator of MASLD progression. Total fat oxidation trended toward a correlation with fibrosis (*r* = 0.49, *P* = 0.056), and RE production was significantly correlated with fibrosis (*r* = 0.50, *P* = 0.047) (Supplemental Figure 6, A and B). Fibrosis was inversely correlated with the rate of anaplerosis relative to the TCA cycle (V_PEPCK_/V_CS_) (*r* = –0.63, *P* = 0.009), and positively correlated with the rate of the TCA cycle relative to EGP (V_CS_/V_EGP_) (*r* = 0.52, *P* = 0.038) (Supplemental Figure 6, C and D). These ratios provide insight into the distribution of REs harvested from fat oxidation, and suggest that with MASLD progression, the sourcing of REs to glucose production may wane. While these fluxes are modeled in participants at a single time point, and thus do not demonstrate longitudinal relationships, these data demonstrate that ketogenesis and total fat oxidation increase in proportion to liver injury, while TCA cycle flux remains relatively stable. These results suggest that ketogenesis and fat oxidation could accelerate during the natural history of MASLD progression to MASH. Given (a) this relationship between ketogenesis and MASLD and (b) the fact that ketogenesis is a strong predictor of total fat oxidation in the liver, we sought to determine the mechanistic connections among these indices using mouse models.

### Loss of HMGCS2 predisposes the liver to steatosis and dysregulated hepatic energy metabolism.

**Figure 4:**
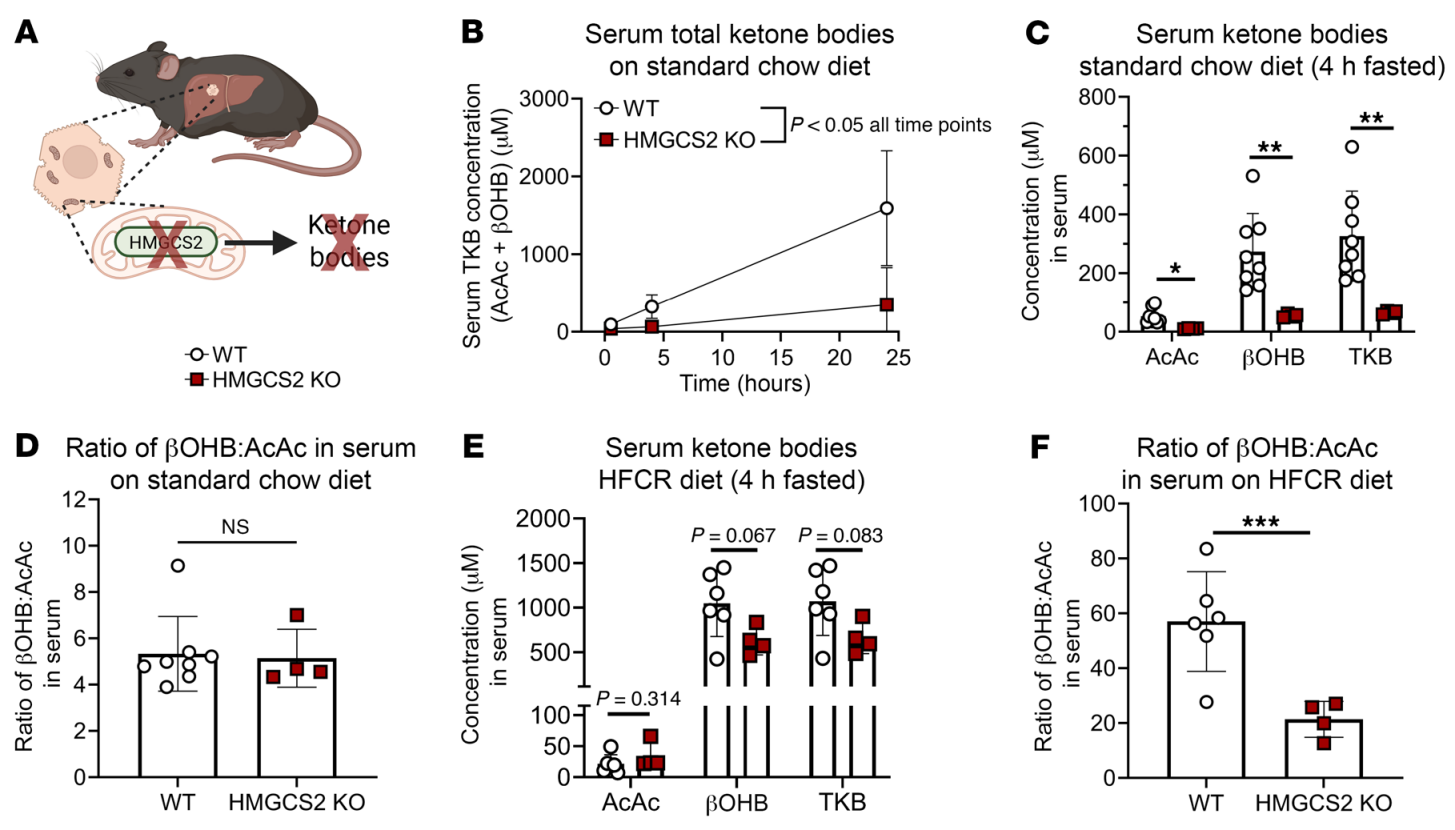
Loss of hepatocyte HMGCS2 impairs fasting ketosis. (**A**) Ketogenesis-null mice were generated by deletion of the gene encoding 3-hydroxymethylglutaryl-CoA synthase 2 (HMGCS2) in hepatocytes. Littermate control (WT) and HMGCS2 hepatocyte-specific knockout (HMGCS2-Liver-KO) male and female mice were maintained on standard chow diet, switched to a high-fat carbohydrate-restricted (HFCR) diet for 1 week, and studied in the random-fed or fasted state. (**B** and **C**) Functional loss of HMGCS2 was confirmed in vivo in chow-fed mice by demonstration that HMGCS2-Liver-KO mice failed to increase fasting total ketone bodies (TKBs) (**B**), marked by a decrease in both AcAc and βOHB (4-hour-fasted) (*n* = 4–8 per group) (**C**). (**D**) Ratio of βOHB to AcAc in 4-hour-fasted WT and HMGCS2-Liver-KO mice fed chow diet (*n* = 4–8 per group). (**E**) Serum TKB analysis of WT and HMGCS2-Liver-KO mice fed HFCR diet (*n* = 4–6 per group). (**F**) The ratio of βOHB to AcAc in WT and HMGCS2-Liver-KO mice fed HFCR diet (*n* = 4–6 per group). Data are expressed as mean ± SD. Statistical differences were determined by Student’s *t* tests and accepted as significant if *P* < 0.05. *P < 0.05, **P < 0.01, ***P < 0.001.

Our previous findings in ex vivo–perfused livers suggested that GNG was impaired in the setting of ketogenic insufficiency (46, 52). Because hepatic GNG is bioenergetically coupled to fat oxidation, this suggested that fat oxidation might be impaired in the absence of ketogenesis. Nonetheless, ketogenesis-insufficient mice maintained euglycemia when fasted, because of compensation from hepatic glycogenolysis and/or extrahepatic GNG (46, 52, 54). To test whether fat oxidation was impaired by ketogenic insufficiency in vivo, we fed littermate control wild-type (WT) and hepatocyte-specific HMGCS2-null (HMGCS2-Liver-KO) mice a high-fat, carbohydrate-restricted (HFCR) diet, and hypothesized that in the absence of dietary carbohydrates, hepatic GNG would fail to support glycemia (Figure 4A). We confirmed loss of HMGCS2 function (48, 55) by demonstrating that chow-fed HMGCS2-Liver-KO mice failed to mount a ketogenic response to fasting but maintained a normal ratio of βOHB to AcAc (Figure 4, B–D) (56). When littermate control WT mice were placed on HFCR diet for 1 week, 4-hour-fasted total ketone bodies were elevated 3.3 ± 0.2–fold, as expected (Student’s *t* test, *P* < 0.001); however, only βOHB was increased, and not circulating AcAc (Supplemental Figure 7A). As a result, the ratio of βOHB to AcAc was increased 10.7 ± 0.2–fold (Supplemental Figure 7B). Unexpectedly, while circulating total ketones in HFCR diet–fed HMGCS2-Liver-KO mice (662 ± 152 μM) trended lower than those in controls (1,071 ± 349 μM) (Student’s *t* test, *P* = 0.08), they were markedly elevated compared with those in chow-fed HMGCS2-Liver-KO mice (66 ± 5 μM) (Figure 4, C and E). Compared with littermate controls maintained on HFCR diet, HMGCS2-Liver-KO mice had a markedly decreased ratio of βOHB to AcAc, but a 3.8 ± 0.1–fold increased total amount of circulating l-βOHB, which does not contribute to mitochondrial NADH/NAD^+^ ratio (Figure 4F and Supplemental Figure 7, C and D). To address whether the ketonemia in HFCR diet–fed HMGCS2-Liver-KO mice was attributable to ketogenesis or to impairments in peripheral ketone body disposal, we quantified static abundance of ketone bodies in liver tissue and found that HFCR diet–fed HMGCS2-Liver-KO livers showed a 69% ± 14% diminution in total ketones (Student’s *t* test, *P* < 0.01), and increased liver l-βOHB, which together support impairment of ketogenesis in livers of HFCR diet–fed HMGCS2-Liver-KO mice (Supplemental Figure 7, E and F).

**Figure 5:**
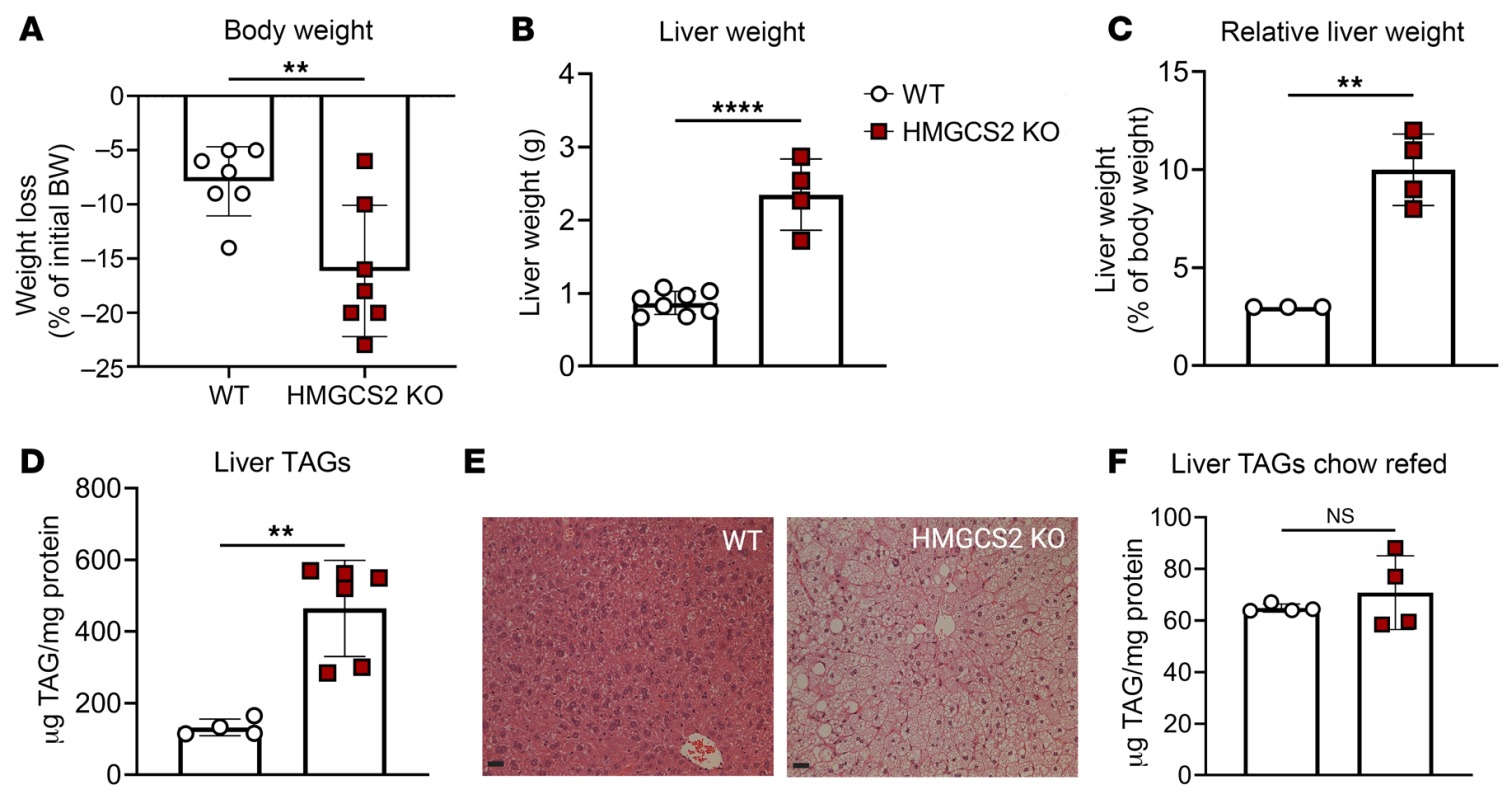
Ketogenic insufficiency induces hepatomegaly and steatosis. Littermate control (WT) and HMGCS2-Liver-KO male and female mice maintained on standard chow diet were switched to HFCR diet for 1 week. (**A**) Net change in body weight after switching of diets (*n* = 7 per group). (**B** and **C**) Total liver weight (grams) (**B**) and relative liver weight (percentage of body weight) (**C**) (*n* = 3–8 per group). (**D** and **E**) Total liver triacylglycerols (TAGs) in random-fed livers quantified colorimetrically (**D**) and shown histologically with H&E stain (**E**). Scale bars: 25 μm. (**F**) Total liver TAGs in HFCR diet–fed mice after 1 week of refeeding of standard chow (*n* = 4 per group). Data are expressed as mean ± SD. Statistical differences were determined by Student’s *t* tests and accepted as significant if *P* < 0.05. **P < 0.01, ****P < 0.0001.

**Figure 6:**
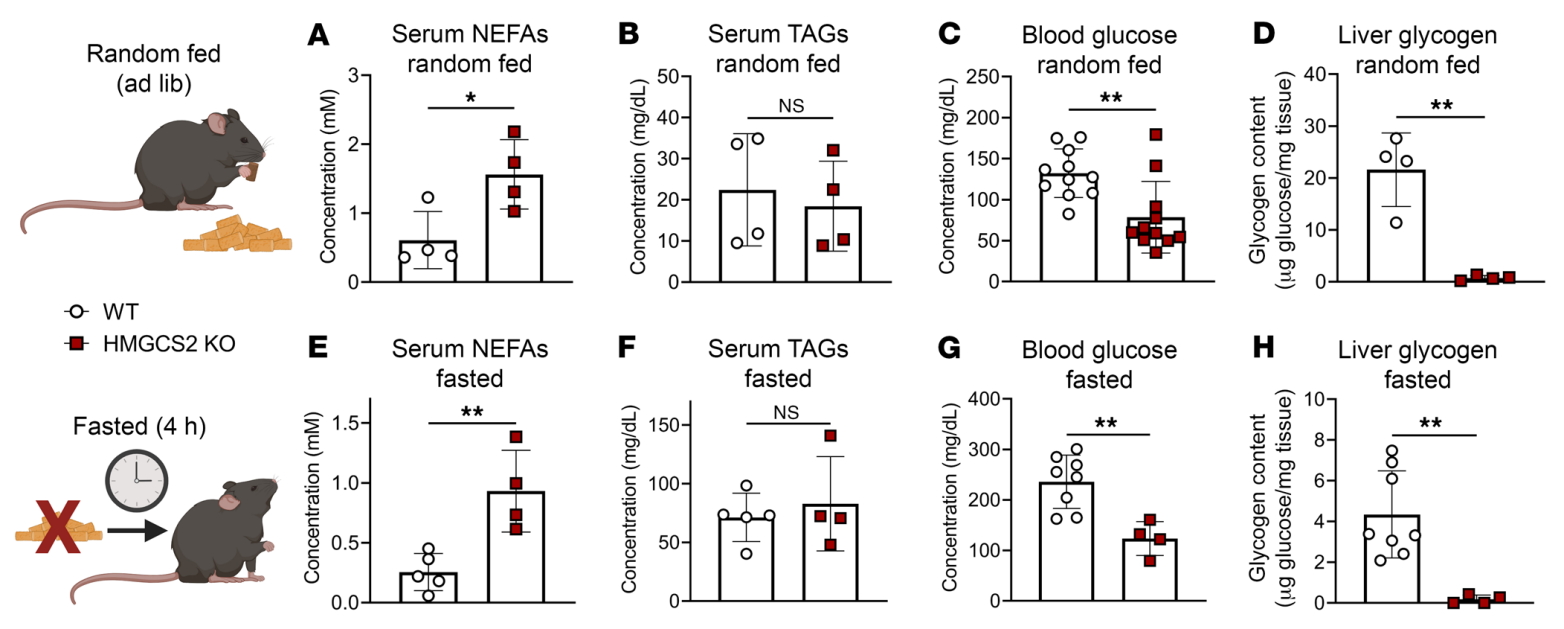
Ketogenic insufficiency induces relative hypoglycemia in HFCR diet–fed mice. Systemic physiological markers of whole-body metabolism in random-fed (**A**–**D**) and 4-hour-fasted (**E**–**H**) male and female littermate control (WT) and HMGCS2-Liver-KO mice, including blood non-esterified fatty acids (NEFAs), TAGs, and glucose. Also included is total liver glycogen content (*n* = 4–11 per group). Data are expressed as mean ± SD. Statistical differences were determined by Student’s *t* tests and accepted as significant if *P* < 0.05. *P < 0.05, **P < 0.01.

To determine the physiological response to HFCR diet in HMGCS2-Liver-KO mice, we quantified gravimetric indices and metabolite concentrations 1 week after transitioning from chow. HMGCS2-Liver-KO mice lost more weight than littermate controls when fed the HFCR diet but developed severe hepatomegaly (Figure 5, A–C). This was accompanied by a 3.5 ± 0.1–fold accumulation of liver TAGs (Figure 5, D and E), which could be reversed by refeeding mice chow diet for 1 week (Figure 5F). In both the random-fed and the 4-hour-fasted state, HMGCS2-Liver-KO HFCR diet–fed mice had elevated plasma NEFAs, no difference in circulating TAGs, decreased blood glucose, and depleted hepatic glycogen (Figure 6, A–H).

**Figure 7:**
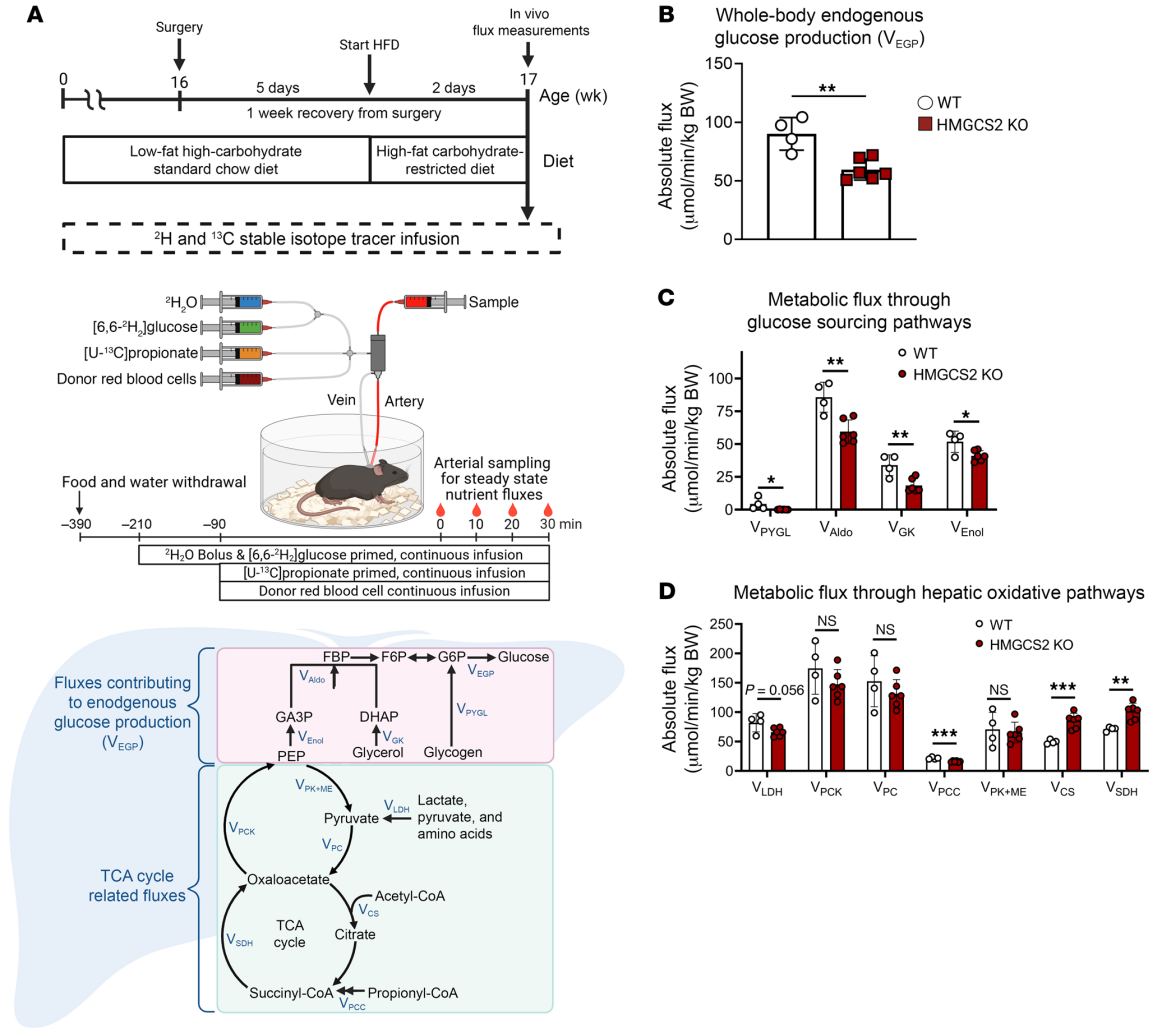
Ketogenic insufficiency diminishes gluconeogenesis in mice. (**A**) Overview of in vivo hepatic flux modeling study design. Top: Fluxes were measured in female littermate control (WT) and HMGCS2-Liver-KO mice. Five days after surgical implantation of indwelling catheters, mice were switched from standard chow to an HFCR diet, and then fluxes were measured after a 2-day acclimation period. Middle: Stable isotope tracers were administered, including [6,6-^2^H_2_]glucose, [U-^13^C_3_]propionate, and heavy water (^2^H_2_O). Red blood cells from donor mice were infused to maintain hematocrit. Samples were collected for analysis during the final 30 minutes after metabolic and isotopic steady state had been reached. Bottom: Diagram of reaction velocities (V) (i.e., fluxes) modeled using a mass isotopomer distribution analysis of derivatized plasma glucose acquired via GC-MS. (**B**) Rates of whole-body endogenous glucose production (V_EGP_) as measured via tracer dilution of [6,6-^2^H_2_]glucose (*n* = 4–6 per group). (**C**) Absolute rates of glucose sourcing pathways, including glycogenolysis (V_PYGL_), total gluconeogenesis (V_Aldo_), and rates of glycerol (V_GK_) and phosphoenolpyruvate (PEP) (V_Enol_) flux to glucose (*n* = 4–6 per group). (**D**) Absolute rates for reactions in the oxidative flux network shown in **A**, including lactate dehydrogenase (LDH; V_LDH_), PEP carboxykinase (PCK; V_PCK_, i.e., total TCA cycle cataplerosis), anaplerosis into the TCA cycle via pyruvate carboxylase (PC; V_PC_) or via propionyl-CoA carboxylase (PCC; V_PCC_), pyruvate cycling as the sum of pyruvate kinase (PK) and malic enzyme (ME) (V_PK+ME_), citrate synthase (CS) flux (V_CS_; i.e., TCA cycle turnover), and succinate dehydrogenase (SDH) flux (V_SDH_) (*n* = 4–6 per group). Data are expressed as mean ± SD. Statistical differences were determined by Student’s *t* tests and accepted as significant if *P* < 0.05. *P < 0.05, **P < 0.01, ***P < 0.001.

To test the hypothesis that impaired hepatic fat oxidation could be linked to diminished glycemia in HFCR diet–fed HMGCS2-Liver-KO mice, we quantified hepatic glucose and associated oxidative fluxes in vivo in conscious, unrestrained mice, using a previously established approach based on gas chromatography–mass spectrometry (GC-MS) (57–59). To accomplish this, vascular catheters were placed in the carotid artery and jugular vein of mice fed chow diet. Five days after surgery, mice were switched to HFCR diet for 2 days. Seven days after surgery, ^2^H_2_O, [U-^13^C_3_]propionate, and [6,6-^2^H_2_]glucose stable isotope tracers were infused, allowing quantification of in vivo fluxes via ^2^H/^13^C metabolic flux analysis (Figure 7A) (51, 58, 59). When fasted, HFCR diet–fed HMGCS2-Liver-KO mice had lower rates of whole-body glucose production (V_EGP_) (59.6 ± 8.2 μmol/min/kg body weight [BW]) compared with littermate controls (90.1 ± 12 μmol/min/kg BW; Student’s *t* test, *P* = 0.003) (Figure 7B). Body weight was not different between groups (data not shown). Both littermate control WT and HMGCS2-Liver-KO mice showed very low rates of glycogenolysis (V_PYGL_, where PYGL stands for glycogen phosphorylase); however, as expected, HFCR diet–fed HMGCS2-Liver-KO mice showed a 31% ± 8% (Student’s *t* test, *P* < 0.01) diminution in total GNG (V_Aldo_, where Aldo stands for aldolase) which was linked to diminished sourcing of glucose from both glycerol (V_GK_) and PEP (V_Enol_) (Figure 7C). GNG fluxes are tightly coupled to the entry (anaplerosis) and exit (cataplerosis) of nutrients through the TCA cycle; thus we next examined glucose-linked hepatic oxidative fluxes. While anaplerosis from propionyl-CoA carboxylase (V_PCC_) was decreased in HMGCS2-Liver-KO mice, most anaplerotic fluxes were not different between genotypes (V_PC_, V_LDH_, and V_PK+ME_, where PC stands for pyruvate carboxylase and LDH stands for lactate dehydrogenase). Cataplerosis (V_PCK_, where PCK stands for PEP carboxykinase) was also similar between HMGCS2-Liver-KO and littermate controls (Student’s *t* test, *P* > 0.05) (Figure 7D). However, HMGCS2-Liver-KO mice showed increased TCA cycle fluxes as evidenced by higher V_CS_ (+72% ± 6%, Student’s *t* test, *P* < 0.001) and V_SDH_ (where SDH stands for succinate dehydrogenase; +42% ± 6%, Student’s *t* test, *P* < 0.01) (Figure 7D). Thus, the V_PCK_/V_CS_ ratio was decreased 50% ± 10% (Student’s *t* test, *P* < 0.001) in HMGCS2-Liver-KO mice, and the V_CS_/V_EGP_ ratio was increased 158% ± 8% (Student’s *t* test, *P* < 0.001) (Supplemental Figure 8A). Moreover, while V_PC_ was unchanged, static concentrations of acetyl-CoA were increased 77% ± 17% (Student’s *t* test, *P* < 0.01) in livers of HFCR diet–fed HMGCS2-Liver-KO mice, indicating an absence of allosteric activation of PC by acetyl-CoA (Supplemental Figure 8B). Moreover, succinyl-CoA and propionyl-CoA were decreased, also suggestive of diminished relative anaplerosis (Supplemental Figure 8B). The total pools of energy adenylates (ATP, ADP, and AMP) were diminished 39% ± 14% in livers of HFCR diet–fed HMGCS2-Liver-KO mice, with no impairment in energy charge (Supplemental Figure 8, C–E). Static liver concentrations of redox metabolites NAD^+^ and NADH were also both diminished 45% ± 13% and 38% ± 14%, respectively, but no difference in liver NAD^+^/NADH ratio was observed in HFCR diet–fed HMGCS2-Liver-KO mice (Supplemental Figure 8, F–H). Together, these findings underscore interconnections among ketogenesis, fat oxidation, anaplerosis/cataplerosis, and EGP. Relative responses of modeled fluxes in HFCR diet–fed HMGCS2-Liver-KO mice quantified in vivo via intravenous infusions were consistent with our prior observations using ex vivo portal vein liver perfusions in chow-fed HMGCS2 knockdown mice, which revealed (a) increased mitochondrial acetyl-CoA, (b) elevated V_CS_, and (c) diminished V_PEPCK_/V_CS_ ratio (46, 52). We next sought to quantify the contribution of ketogenesis to hepatic fat oxidation, and how this influences the course of MASLD-MASH.

### Ketogenic insufficiency impairs total hepatic fat oxidation.

**Figure 8:**
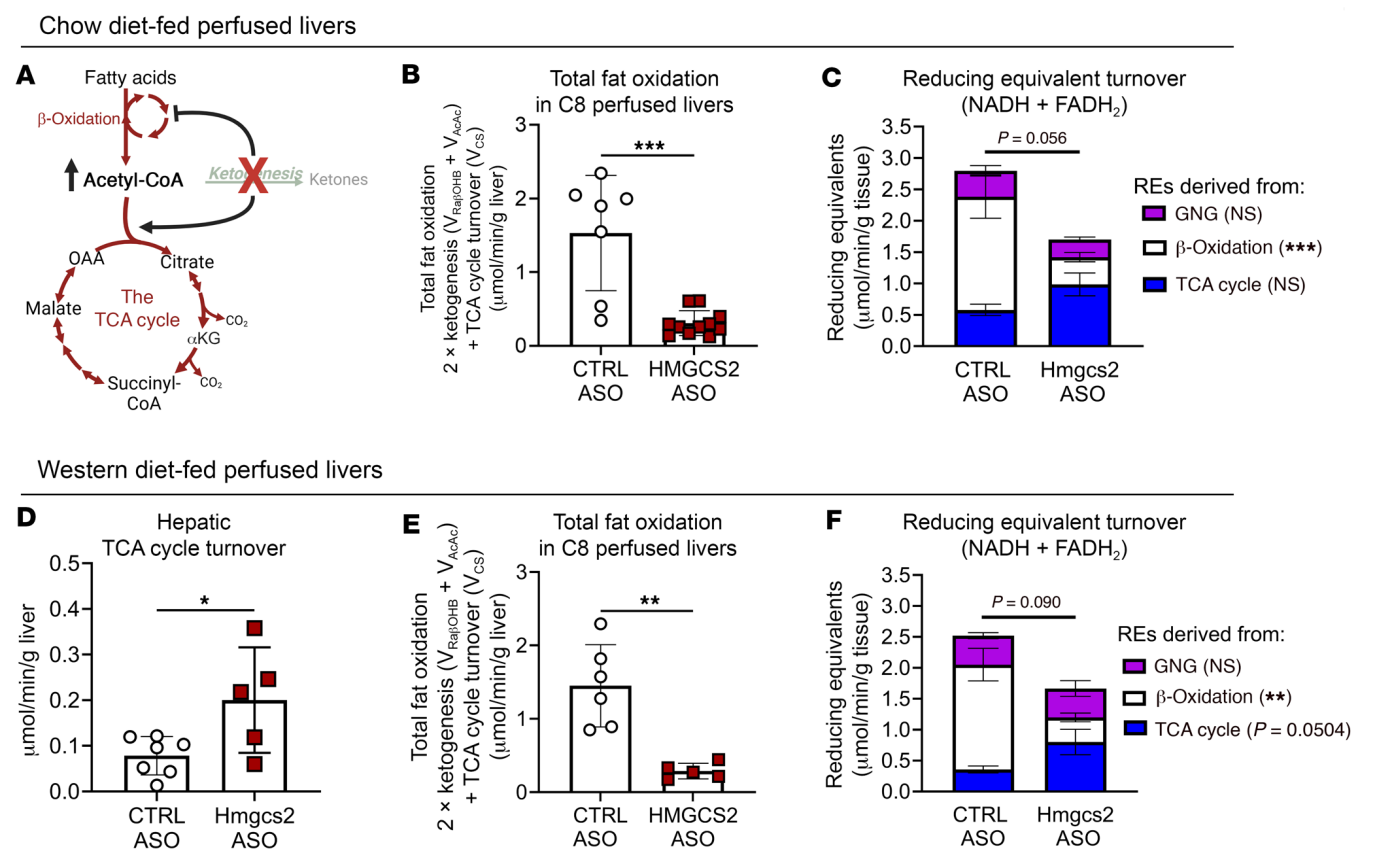
Ketogenic insufficiency impairs fat oxidation in perfused livers. (**A**) Prior studies have demonstrated that ketogenesis insufficiency induced by loss of HMGCS2 causes accumulation of mitochondrial acetyl-CoA and acceleration of the TCA cycle. Livers of male mice treated with scrambled control antisense oligonucleotide (ASO) or mouse *Hmgcs2* ASO were perfused with octanoate (C8) and oxidative fluxes quantified using ^2^H/^13^C stable isotope tracing. (**B** and **C**) Total fat oxidation quantified as the summation of (2 × ketogenesis) + TCA cycle turnover (**B**), and reducing equivalent (RE) turnover (NADH + FADH_2_) broken down into REs from gluconeogenesis (GNG), β-oxidation, and the TCA cycle (**C**), in perfused livers from control and *Hmgcs2* ASO mice on chow diet (*n* = 10–11 per group). Fat oxidation was also studied in livers perfused ex vivo with C8 from control and *Hmgcs2* ASO–treated mice maintained on a 42% high-fat high-sucrose Western diet (WD) for 8 weeks. (**D**–**F**) TCA cycle turnover (**D**), total fat oxidation (**E**), and RE production rate (NADH + FADH_2_) (**F**) in perfused livers from control and *Hmgcs2* ASO–treated mice on WD (*n* = 5–6 per group). Data are expressed as mean ± SD. Statistical differences were determined by Student’s *t* tests or 2-way ANOVA and accepted as significant if *P* < 0.05. *P < 0.05, **P < 0.01, ***P < 0.001.

To determine whether ketogenic insufficiency impaired hepatic fat oxidation (Figure 8A), including those components accounted for by ketogenesis and V_CS_, we returned to ketogenesis-insufficient ex vivo–perfused livers in carbohydrate-replete settings. Our group previously characterized the HMGCS2 antisense oligonucleotide (ASO) knockdown liver and showed (a) abrogation of liver HMGCS2 protein, but its preservation in non-hepatic tissues; (b) abrogation of fasting-induced ketogenesis; (c) elevation of V_CS_ and V_CS_/V_GNG_ ratio; and (d) increased predisposition to steatotic liver injury (46, 47, 52, 56). Compared with control mice receiving scrambled ASO, the livers of chow-fed ketogenesis-insufficient mice exhibited an 80% ± 24% diminution in total fat oxidation (Student’s *t* test, *P* < 0.001), a 76% ± 24% impairment in REs generated from β-oxidation, and a strong trend toward a decrease in total RE production rate (39% ± 23% Student’s *t* test, *P* = 0.056), despite a trend toward a 70% ± 23% increase in TCA cycle–sourced REs (Student’s *t* test, *P* = 0.1108) (Figure 8, B and C). There were no differences in REs generated through GNG. Concordantly, in livers of HMGCS2-knockdown mice fed a sucrose-enriched Western diet (WD) with 42% kcal from fat for 8 weeks, we observed that V_CS_ was increased 155% ± 30% (Student’s *t* test, *P* = 0.03), and thus REs produced by the TCA cycle strongly trended toward a greater-than-2-fold increase (Student’s *t* test, *P* = 0.05), suggesting that loss of ketogenesis stimulates terminal fat oxidation in the TCA cycle in WD-fed mice (Figure 8, D–F). Nonetheless, the data support the hypothesis that the rate of total fat oxidation is impaired by ketogenic insufficiency.

**Figure 9:**
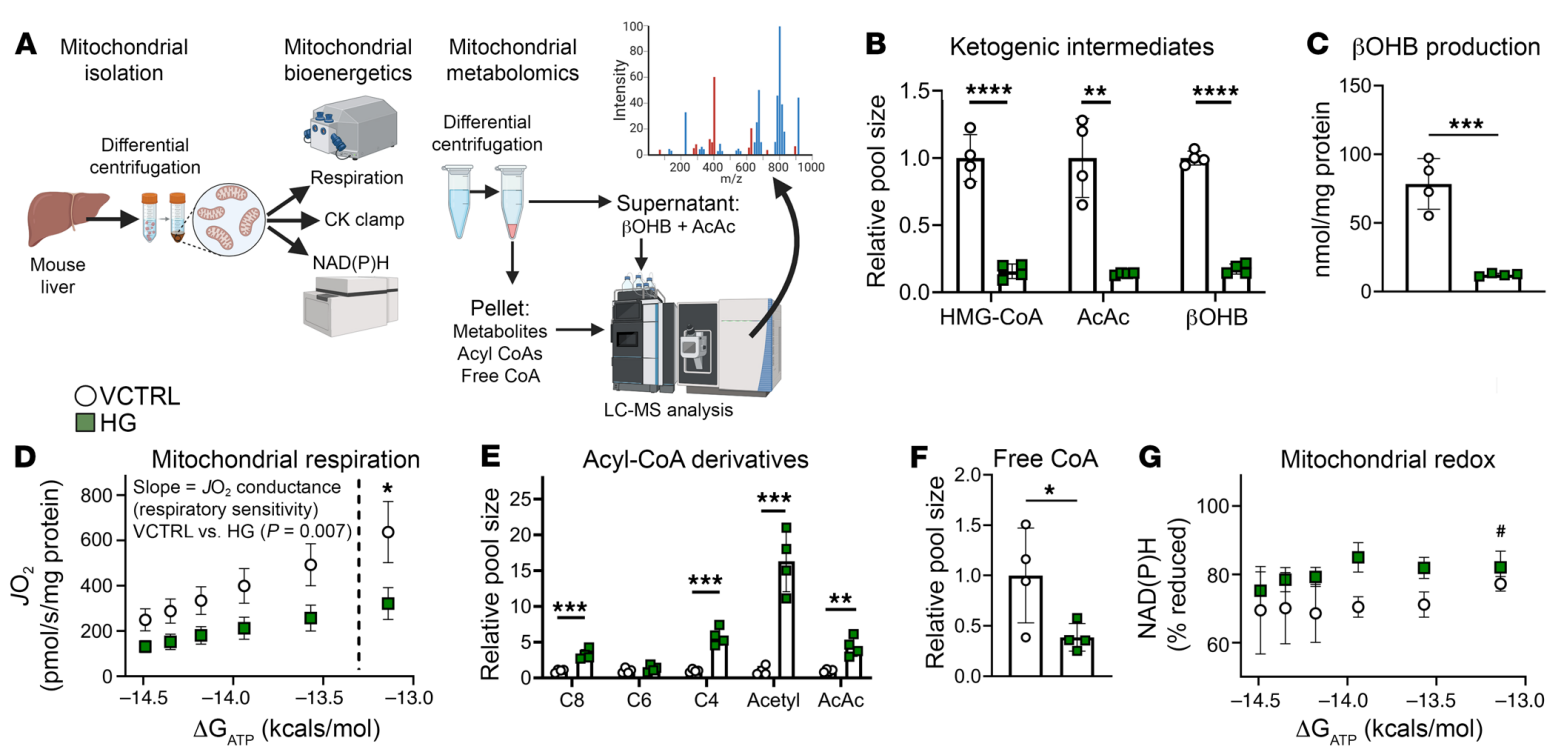
Ketogenic insufficiency impairs fat oxidation in isolated mitochondria. (**A**) Mitochondrial diagnostics workflow. Liver mitochondria isolated by differential centrifugation and fueled by palmitoyl-l-carnitine plus α-ketoglutarate were used to assess the effects of hymeglusin (HG; HMGCS inhibitor) versus vehicle control (VCTRL) on βOHB production, respiratory kinetics, and redox potential during a creatine kinase (CK) energetic clamp, and on metabolite abundance after CK clamp. (**B** and **C**) Relative abundance of HMG-CoA, AcAc, and βOHB in mitochondria (**B**) and βOHB production measured in supernatant collected after CK clamp (**C**) (*n* = 4 per group). (**D**) Respiration (*J*O_2_) plotted as a function of energy demand (ΔG_ATP_ [kcal/mol]), with respiratory sensitivity (i.e., *J*O_2_ conductance) measured as the slope of the curve (*n* = 4 per group). Results of Student’s *t* test of slopes between VCTRL and HG are shown on the graph. (**E** and **F**) Relative abundance of acyl-CoA species (**E**) and free coenzyme A (**F**) in mitochondria (*n* = 4 per group). (**G**) Redox potential [NAD(P)H percentage reduction] plotted as a function of energy demand (ΔG_ATP_ [kcal/mol], *n* = 4 per group). Pool sizes are from mitochondria after incubation at a fixed energy demand (ΔG_ATP_ = –13.94 kcal/mol). Data are expressed as mean ± SD. Statistical differences were determined by Student’s *t* tests or 2-way ANOVA and accepted as significant if *P* < 0.05. *P < 0.05, **P < 0.01, ***P < 0.001, ****P < 0.0001, ^#^significant by 2-way ANOVA (*P* < 0.05).

To address underlying mechanisms for impaired fat oxidation, we isolated mitochondria from control C57BL/6NJ mouse livers and performed mitochondrial stress testing using our standard creatine kinase (CK) clamp protocol, allowing quantification of respiratory flux (*J*O_2_) across variations in energy demand (ΔG_ATP_) (Figure 9A) (60). Treatment of mitochondria with hymeglusin (HG), a small-molecule inhibitor of both HMGCS isoforms (HMGCS1 is a cytoplasmic enzyme and is thus not included in our mitochondrial preparations), showed the expected diminution in HMG-CoA as well as AcAc and βOHB within mitochondria, coinciding with abrogated βOHB production rates in mitochondria stimulated with palmitoyl-l-carnitine plus α-ketoglutarate (Figure 9, B and C) (61, 62). With increasing energy demand, *J*O_2_ rates increased with a diminished slope in HG-treated mitochondria, indicating an overall respiratory impairment due to ketogenic insufficiency (Figure 9D). This coincided with an accumulation of C2–C8 acyl-CoA species, and a depletion in free CoA (Figure 9, E and F). Finally, mitochondrial redox was more reduced in HG-treated mitochondria [NAD(P)H, % reduced, Figure 9G). While this result may appear to contradict the normal redox ratio observed in livers of HFCR diet–fed HMGCS2-Liver-KO mice (Supplemental Figure 8H), static concentrations of total redox nucleotides may not reflect variations in cycling enzymatically bound pools (53, 63). Taken together, these findings show that ketogenic insufficiency impairs total hepatic fat oxidation in vivo, ex vivo, and in isolated mitochondria. One potential contributor to this impairment is free CoA trapping, which impedes procession through β-oxidation and the TCA cycle; this is supported by our prior findings (46). A second contributor to diminished fat oxidation rates is reduced mitochondrial matrix redox potential in the setting of ketogenic insufficiency, captured in this flux-based assay that quantifies redox nucleotide turnover among actively cycling enzymatic pools.

### Loss of mitochondrial βOHB dehydrogenase impairs hepatic fat oxidation but does not provoke liver injury.

**Figure 10:**
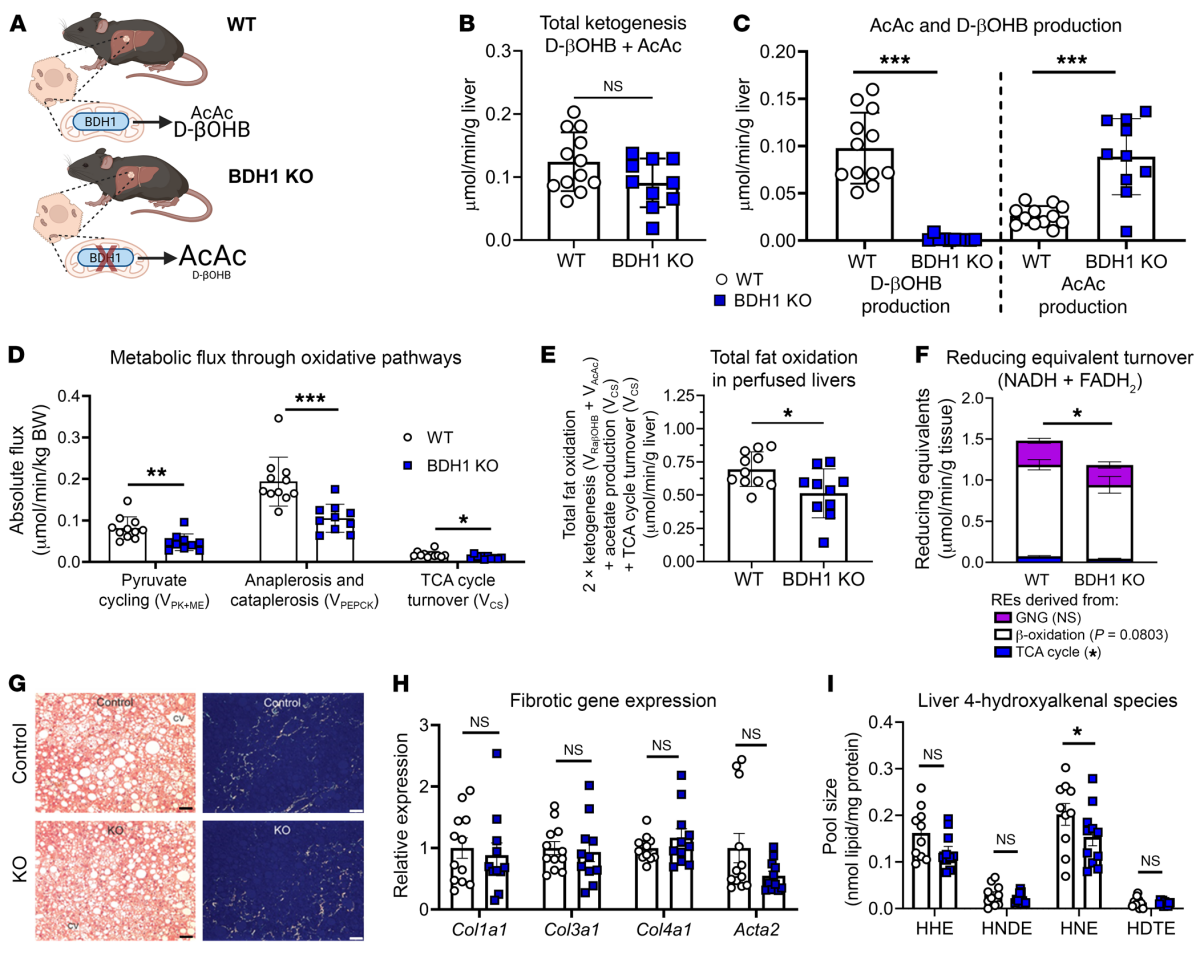
Loss of hepatocyte BDH1 impairs total hepatic fat oxidation but does not exacerbate HFD-induced liver fibrosis. (**A**) Male hepatocyte-specific BDH1-null (BDH1-Liver-KO) and littermate control (WT) mice were maintained on standard chow diet, then were switched to a 42% high-fat high-sucrose WD for 16 weeks, after which oxidative fluxes were studied in livers perfused ex vivo with long-chain fatty acids. (**B** and **C**) Ketone body production (**B**) and AcAc and βOHB production (**C**) in livers of WT and BDH1-Liver-KO mice (*n* = 10–11 per group). (**D**) Absolute rates of oxidative pathways, including pyruvate cycling (V_PK+ME_), total anaplerosis and cataplerosis (V_PEPCK_), and TCA cycle turnover (V_CS_) (*n* = 10–11 per group). (**E** and **F**) Total fat oxidation calculated at (2 × ketogenesis) + acetogenesis + TCA cycle turnover (**E**), and reducing equivalent (RE) turnover (NADH + FADH_2_) broken down into REs generated from gluconeogenesis (GNG), β-oxidation, and the TCA cycle (**F**) (*n* = 10–11 per group). (**G**–**I**) Liver H&E and Picrosirius red histological staining (**G**), gene expression for fibrotic gene markers (**H**), and absolute levels of lipid peroxide 4-hydroxyalkenal species (**I**) in livers of WT and BDH1-Liver-KO mice maintained on 42% kcal fat WD (*n* = 10–12 per group). Scale bars: 25 μm in H&E images, 100 μm in Picrosirius red images. HHE, 4-hydroxy-2-hexenal; HNDE, 4-hydroxynona-2E, 6Z-dienal; HNE, 4-hydroxy-2-nonenal; HDTE, 4-hydroxy-2E, 6Z, 8Z-decatrienal. Data are expressed as mean ± SD. Statistical differences were determined by Student’s *t* tests and accepted as significant if *P* < 0.05. *P < 0.05, **P < 0.01, ***P < 0.001.

Ketogenic insufficiency via loss of HMGCS2 function causes steatotic liver injury (45–49). Our findings show that loss of HMGCS2 diminishes rates of liver fat oxidation in a manner correlated with more reduced mitochondrial redox potential. To interrogate the role of the ketogenic reaction directly regulating mitochondrial redox potential, catalyzed by mitochondrial βOHB dehydrogenase (BDH1) (which converts AcAc and NADH to βOHB and NAD^+^) in (a) total hepatic fat oxidation and (b) the response to steatotic liver injury, we studied mice in which BDH1 had been deleted specifically in hepatocytes (BDH1-Liver-KO mice) (Figure 10A). Our prior studies demonstrated impairments of oxidative metabolism in BDH1-Liver-KO mice on chow diet (53). Here, we fed mice 42% kcal fat WD for 16 weeks. As expected, circulating βOHB and total ketones were diminished after an 18-hour fast (Supplemental Figure 9A). Body weights, liver weights, and blood glucose were unchanged (Supplemental Figure 9, B–D). Livers were then perfused ex vivo with a physiologically relevant mixture of long-chain fatty acids conjugated to bovine serum albumin. While there was no significant diminution in total ketogenesis in livers of BDH1-Liver-KO mice, all ketone bodies emerged as AcAc, with no hepatic βOHB production (Figure 10, B and C). V_EGP_ was diminished 26% ± 13% (Student’s *t* test, *P* = 0.0390) in livers of BDH1-Liver-KO mice, which coincided with a 48% ± 12% and 30% ± 13% decrease in sourcing of glucose from both PEP (Student’s *t* test, *P* < 0.001) and glycogen (Student’s *t* test, *P* = 0.04), respectively (Supplemental Figure 9, E–G). Consistent with our previous findings observed in chow-fed mice, the rates of pyruvate cycling (V_PK+ME_), anaplerosis and cataplerosis (V_PEPCK_), and TCA cycle turnover (V_CS_) were all suppressed 42% ± 16% (Student’s *t* test, *P* = 0.0043), 15% ± 13% (Student’s *t* test, *P* = 0.0005), and 39% ± 17% (Student’s *t* test, *P* = 0.0160), respectively, in livers of BDH1-Liver-KO mice on WD (Figure 10D). Furthermore, total fat oxidation was decreased 26% ± 12% (Student’s *t* test, *P* = 0.0170), and RE production rate was decreased 20% ± 10% (Student’s *t* test, *P* = 0.0259) (Figure 10, E and F), demonstrating that loss of the NAD^+^ regenerating step of ketogenesis alone is sufficient to impair overall fat oxidation in the liver.

To determine whether the diminution in hepatic fatty acid (FA) oxidation in livers of BDH1-Liver-KO mice was linked to increased steatosis or injury in mice fed a 42% kcal fat WD for 16 weeks, we performed histological and molecular analyses. H&E staining showed normal lipid droplet storage, and no evidence of increased fibrosis via Picrosirius red staining in comparison with controls (Figure 10G). Gene expression biomarkers of fibrosis (*Col1a1*, *Col3a1*, *Col4a1*, *Acta2*) were unchanged and quantities of tissue lipid peroxides were not increased in livers of BDH1-Liver-KO mice (Figure 10, H and I). Maintenance of independent cohorts of BDH1-Liver-KO and littermate control mice on a 60% kcal high-fat diet (HFD) for 16 weeks also showed no evidence of increased steatosis, inflammation, or fibrosis — with a trend toward a diminution in fibrosis in livers of BDH1-Liver-KO mice (56% ± 30%, Student’s *t* test, *P* = 0.06) (Supplemental Figure 10, A–C). Moreover, gene expression biomarkers of fibrosis and inflammation were diminished in livers of BDH1-Liver-KO mice fed a 60% HFD (Supplemental Figure 10C). Lastly, we induced liver injury in mice by feeding a fibrogenic choline-deficient, methionine-limited high-fat (62% kcal) diet for 16 weeks, but did not detect worsened steatosis, injury, fibrosis, or inflammation in BDH1-Liver-KO mice (Supplemental Figure 10, D and E). Collectively, these findings demonstrate (a) that ketogenesis is required to maintain hepatic fat oxidation and its coupling to V_EGP_, with loss of either HMGCS2 or BDH1 impairing hepatic fat oxidation; and (b) that the impairment in fat oxidation does not fully explain why loss of HMGCS2 predisposes the liver to HFD-induced liver injury.

## Discussion

The natural history of MASLD-MASH proceeds through derangements of both liver-autonomous and systemic metabolic pathways, among many cell types. Characteristic metabolic signatures include imbalanced lipid synthesis (de novo lipogenesis and TAG synthesis) and disposal (mitochondrial fat oxidation and TAG secretion as VLDL). While most studies concur that de novo lipogenesis (DNL) is augmented, and VLDL secretion does not match the rate of lipid accumulation, controversy surrounds variations in the rate of mitochondrial fat oxidation and whether variations in this rate have a causal role. This impacts the selection of therapeutic molecular targets worthy of pursuit. In this study, we quantified hepatic oxidative fluxes in humans with histologically confirmed MASLD-MASH and demonstrated that the histopathological degree of liver injury was positively correlated with ketogenesis and total fat oxidation but not TCA cycle turnover. Use of mouse models suggested a driver role of ketogenesis in overall hepatic fat oxidation and a causal role in preventing liver injury, as elimination of HMGCS2 diminished hepatic fat oxidation. This decrement correlates with increased steatotic liver injury, as shown in other studies in the setting of ketogenic insufficiency (45–49). Thus, the increased ketogenesis observed in patients with higher NAS may serve a compensatory mechanism, increasing fat disposal through oxidation, in the absence of increases in the rates of TCA cycle–linked terminal oxidation. This position is challenged, however, by the results observed in mice lacking hepatocyte BDH1, whose deficiency also diminishes hepatic fat oxidation rates but is not associated with worsened steatotic liver disease. In fact, signatures of a modestly adaptive response to diminished hepatic fat oxidation are evident in the livers of BDH1-Liver-KO mice. Taken together, these results suggest that while ketogenic sufficiency may be important to slow the progression of MASLD, the salutary mechanism conferred by ketogenesis may not only relate to maximizing hepatic fat oxidation. Therefore, approaches aimed primarily at stimulating hepatic fat oxidation may not ameliorate the course of commonly observed MASLD, and could exacerbate liver injury.

Observations of quantified rates of hepatic fat oxidation in humans with MASLD, relative to steatosis-free but weight-matched controls, have been disparate (22–25, 30–37, 50, 64, 65). Much of this may relate to the populations studied — including (a) the stage of progression; (b) the degree of adiposity and insulin resistance; (c) whether or not the participant was weight neutral; (d) the genotype (e.g., *PNPLA3*); and (e) whether the participant was fed, fasting, or undergoing a clamp study — and to the specific methodology employed. While in vivo hepatic oxidative fluxes have been quantified in humans with uncomplicated MASLD, and isolated mitochondrial function has been measured in liver biospecimens harvested from participants with MASH, in vivo hepatic oxidative fluxes have not been previously quantified in humans with histologically confirmed MASH. In well-performed studies by the Burgess and Browning group, ketogenesis negatively correlated with worsening hepatic steatosis in fasting overweight and obese participants with BMI ≤ 35 (22, 37). Moreover, circulating ketones and expression levels of ketogenic enzymatic machinery decreased with worsening NAS in a bariatric surgery cohort studied by the Rector group, but no study has tested whether endogenous ketogenesis correlates with histopathologically confirmed liver injury (33, 34). Consistent with our findings, a recent study in non-obese participants with MASLD by the Petersen and Shulman group showed that participants with compounding cardiometabolic disturbances have increased rates of βOHB turnover in comparison with participants with simple steatosis alone, or with healthy livers, but liver biopsies were not available in that study (27). The cohort studied in our experiments reflects a population with advanced obesity at elevated risk of progression from MASH to cirrhosis, and among the metabolic fluxes modeled, the only direct correlate of NAS was endogenous ketogenesis. We propose that the escalation of TCA cycle flux early in the progression of MASLD may become attenuated with further progression, and indeed, the two participants we studied with the greatest V_CS_ (>12 μmol/min/kg LBM) had NAS 4, and the V_CS_ of those exhibiting a NAS greater than 4 did not exceed 10 μmol/min/kg LBM. Thus, with respect to variations of TCA cycle flux, our data are generally consistent with those of the Burgess and Browning group (22, 25). Moreover, while impaired fasting ketogenesis was reported in a relatively leaner population of participants than those studied here, the same subjects showed elevated ketosis in the postabsorptive state and during a clamp (22). Taken together, the aggregate data in both humans and mouse models suggest that ketogenesis may serve an adaptive counterregulatory role, especially with further MASH progression.

Our findings in mouse models of altered ketogenesis suggest that impairments in hepatic fat oxidation in ketogenic insufficiency do not relate in a direct manner to liver injury, implying that the link between ketogenesis and MASLD-MASH progression involves attributes of ketogenesis beyond its contribution to hepatic fat oxidation. Concordance between our observations in modeled hepatic fluxes of the human participants with MASH and mouse models of ketogenic insufficiency strongly substantiates this claim. In addition to the direct association between ketogenesis and human NAS score, increasing histopathological evidence of fibrosis was correlated inversely with V_PEPCK_/V_CS_ ratio and directly with V_CS_/V_EGP_ ratio (Supplemental Figure 6, C and D), suggestive of decoupling between TCA cycle flux and sourcing of its derived REs to GNG with increasing liver injury. Similarly, V_PCK_/V_CS_ ratio was decreased in steatosis- and injury-prone HMGCS2-Liver-KO mice, and V_CS_/V_EGP_ ratio was increased (Supplemental Figure 8A). Conversely, this imbalance was not observed in livers of BDH1-Liver-KO mice, whose V_PEPCK_, V_EGP_, and V_CS_ were all coordinately diminished (Figure 10D). Alternatively, and not mutually exclusively, mechanisms linked to the increase in AcAc production in livers of BDH1-Liver-KO mice could explain why the loss of BDH1 protects the liver from injury (47).

Recently, we reported a role for ketogenesis in supporting FA elongation and polyunsaturated fatty acid (PUFA) homeostasis in the liver (48). We demonstrated that (a) ketogenesis furnishes hepatocytes with malonyl-CoA for FA biosynthesis, via both DNL and FA elongation, and (b) incorporation of ketone body–derived carbon into FA elongation requires the cytosolic enzyme acetoacetyl-CoA synthetase (AACS), through a metabolic pathway independent of ketone-sourced DNL. Furthermore, we demonstrated that loss of HMGCS2 impairs FA elongation and diminishes hepatic PUFAs, suggesting the ketogenesis could influence mitochondrial function and VLDL secretion through PUFA-dependent mechanisms, though these possibilities have not yet been tested. It is tempting to speculate that the pathway linking ketogenesis to PUFA biosynthesis explains the protective nature of ketogenesis in MASLD-MASH progression, and the observation that AcAc production is increased in the absence of BDH1 suggests that FA elongation and PUFA biosynthesis could be stimulated in a substrate-driven manner. PUFAs inhibit DNL in the liver via inhibition of SREBP1c-mediated gene transcription (66–71), and therefore the rise in ketone body–sourced PUFAs in the BDH1 knockout could drive both acute and chronic inhibition of DNL. This hypothesis is further supported by the observation that loss of HMGCS2 induces a DNL gene expression profile (46, 48).

Collectively, these findings in humans with MASH and in ketogenesis-insufficient mice suggest that ketogenesis may compensate in a fat-overloaded, progressively injured liver, thereby maintaining rates of fat oxidation, but perhaps just as importantly by burning fat through a metabolic pathway that confers additional benefits that terminal oxidation in the TCA cycle may not bestow. The specific regulatory mechanisms that coordinate the fate of β-oxidation–derived acetyl-CoA through the TCA cycle or ketogenesis remain incompletely understood. Classical mechanisms include oxaloacetate availability, allosteric regulation of citrate synthase, NADH redox potential, and ΔG_ATP_. Physiological variation in the abundance of HMGCS2 protein could serve as a regulatory mechanism, but this has not been definitively proven. Our observation that ketogenesis did not correlate with circulating NEFAs supports the notion that liver-autonomous mechanisms mediate the acceleration in ketogenesis in the setting of worsened liver injury. Irrespective, the pathways through which ketogenesis and ketone bodies could influence DNL, PUFA synthesis, VLDL secretion, fat oxidation, and canonical signaling pathways, open opportunities to understand the therapeutic potential for ketone bodies to ameliorate MASLD.

### Limitations.

The human studies are limited by a small sample size (16 participants), and were predominantly female (15 female, 1 male). While all participants were insulin resistant, only one participant had diabetes, so it is not possible to make conclusions relevant to the scope of MASLD within diabetes. Two independent experimental approaches were leveraged to delete HMGCS2 protein in mice, but the concordance in phenotypes across these models supports the rigor of our findings. Using metabolic flux modeling, we demonstrated that disruption of ketogenesis impaired overall hepatic fat oxidation, which was quantified via an indirect flux modeling–based assay predicated on the assumption that mitochondrial acetyl-CoA turnover at steady state is mathematically equivalent to 2 × total ketogenesis (V_RaTKB_) + TCA cycle turnover (V_CS_) + acetogenesis (V_acetate_). A limitation of this approach is the assumption that fat oxidation is primarily sourced from exogenous fat during perfusions, and the assumption that octanoate sources little acetate. The findings, however, are reinforced by the concordance among all the fluxes modeled across all orthogonal methodologies employed. Collectively, an impairment in fat oxidation was consistently observed in the setting of ketogenic insufficiency, as shown across various models, including: (a) in vivo HFCR diet–fed HMGCS2-Liver-KO mice; (b) ex vivo–perfused livers from HMGCS2 ASO–knockdown mice maintained on either chow or WD; (c) ex vivo–perfused livers from BDH1-Liver-KO mice maintained on chow or WD; and (d) isolated mitochondria treated with HG. Collectively, these data strongly support the hypothesis that ketogenesis plays an integral role in regulating fat oxidation. A modest limitation of in vivo ketogenesis measurements in humans was that ketogenesis was modeled as a single pool, with V_RaAcAc_ calculated from V_RaβOHB_ and the ratio of βOHB to AcAc. Future experiments will infuse independent [^13^C]AcAc and [^13^C]βOHB tracers to directly measure V_RaAcAc_ and interconversion of AcAc and βOHB through BDH1 via a 2-pool model.

## Methods

See Supplemental Methods for detailed methodology and calculations.

### Sex as a biological variable.

Males and females were studied, as delineated in the figure legends. However, sex as a biological variable was not addressed.

### Human MASLD-MASH study design.

Sixteen participants with BMI ≥ 35 and liver PDFF greater than 5% were recruited. Insulin sensitivity and body composition were assessed via frequently sampled intravenous glucose tolerance test (FSIVGTT) and DXA imaging, respectively, followed by in vivo hepatic oxidative flux measurements. Before flux measurements, a liver biopsy was obtained for determination of the NAFLD activity score (NAS).

### Stable isotope delivery in humans.

Fasted participants were provided with 3 equal doses of 70% deuterated water (^2^H_2_O, 5 g/kg body water), along with 0.5% ^2^H_2_O available for ad libitum consumption to enrich body water, and 2 doses of oral [U-^13^C_3_]propionate (300 mg/dose) to label TCA cycle intermediates for profiling of hepatic mitochondrial fluxes. Intravenous stable isotope infusion began with [3,4-^13^C_2_]glucose (0.563 mg/kg BW bolus, immediately followed by a 2-hour infusion [0.00563 mg/min/kg BW]) and d-[U-^13^C_4_]βOHB (1 mg/kg BW bolus, immediately followed by 2-hour infusion [0.01 mg/min/kg BW]). At the end of the 2-hour infusion, about 50 mL of blood was drawn and centrifuged to isolate plasma, and then aliquots were frozen at –80°C until further analysis.

### ^2^H/^13^C NMR hepatic flux modeling.

The positional ^13^C and ^2^H isotopomers of plasma glucose encode quantitative information about hepatic biochemical pathways, thus allowing for noninvasive modeling of hepatic metabolism in vivo. Direct quantification of the positional isotopomer populations of plasma glucose was achieved using ^13^C and ^2^H NMR, after derivatization into 1,2-isopropylidene glucofuranose (monoacetone glucose [MAG]) as previously described (25, 36, 51, 52).

### Quantification of whole-body βOHB turnover.

βOHB turnover was calculated at metabolic and isotopic steady state from the dilution of intravenously infused d-[U-^13^C_4_]βOHB using the equation:

 (Equation 1)
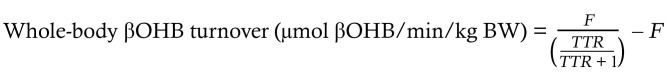


[^12^C]βOHB and [U-^13^C_4_]βOHB were quantified using UHPLC–tandem MS (UHPLC-MS/MS), and then the tracer/tracee ratio ([U-^13^C_4_]βOHB/[^12^C]βOHB) in plasma and the tracer infusion rate (*F*) were used to calculate whole-body βOHB turnover rates. Because the liver is the primary site of systemic ketone body production, whole-body βOHB turnover at steady state is mathematically equivalent to hepatic endogenous βOHB production rates (V_RaβOHB_). After flux measurements, turnover rates were corrected for LBM from DXA imaging, and expressed as μmol βOHB turned over/min/kg LBM.

### Quantification of whole-body glucose turnover.

Glucose turnover was calculated at metabolic and isotopic steady state from the dilution of intravenously infused [3,4-^13^C_2_]glucose using the equation:

 (Equation 2)
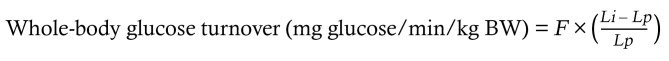


where *F* is tracer infusion rate, *L_p_* is fractional ^13^C enrichment of the plasma glucose pool measured via ^13^C NMR, and *L_i_* is the enrichment of infused glucose, assumed to be 99%. In the fasted state the majority (>90%) of whole-body glucose production is derived from the liver; therefore total glucose turnover rates are mathematically equivalent to total hepatic EGP rates (V_EGP_). Using the DXA scan information, final flux units were expressed as μmol glucose turned over/min/kg LBM.

### Quantification of hepatic glucose sourcing fluxes.

Using the ^2^H enrichment patterns encoded in plasma glucose, the fractional contribution of various glucose sourcing pathways was determined, then multiplied by total EGP (V_EGP_) to calculate absolute flux through each hepatic glucose sourcing pathway using the following equations:

 (Equation 3)
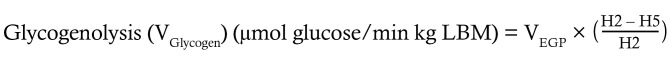


 (Equation 4)
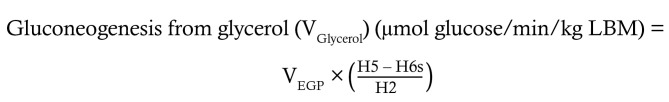


 (Equation 5)
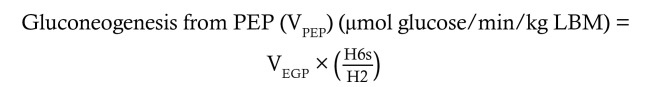


where H2, H5, and H6s correspond to the relative ^2^H enrichment at the 2, 5, and 6s carbons of MAG.

### Quantification of hepatic oxidative fluxes.

The rate of hepatic TCA cycle turnover, anaplerosis, cataplerosis, and pyruvate cycling can be modeled in the liver using the ^13^C isotopomers that arise at C2 of plasma glucose from orally administered [U-^13^C_3_]propionate using the following equations:

 (Equation 6)
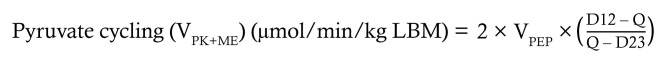


 (Equation 7)
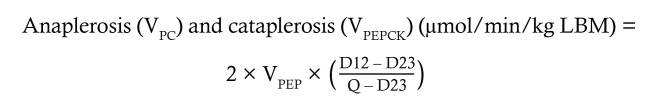


 (Equation 8)
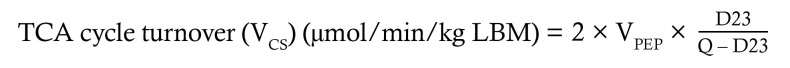


where D12 represents the doublet arising when C1 and C2 of glucose are labeled, D23 represents the doublet arising when C2 and C3 are labeled, and Q is the quartet arising when C1, C2, and C3 are all labeled.

### Quantification of hepatic fat oxidation rates.

In the fasted liver, β-oxidation–derived acetyl-CoA is (a) principally converted to ketone bodies, which are largely released from the liver; (b) terminally oxidized in the TCA cycle; or (c) secreted as acetate. By assuming that the liver is in a metabolic steady state, the rate of acetyl-CoA production via β-oxidation can be assumed to be equal to the rate of acetyl-CoA disposal, thereby allowing total fat oxidation rates to be indirectly quantified as the sum of all disposal routes for hepatic acetyl-CoA as follows:

 (Equation 9)
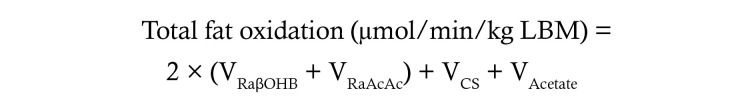


Fat oxidation is expressed in μmol of acetyl-CoA turned over/min/kg LBM. V_RaβOHB_ was measured via a tracer dilution of intravenously infused d-[U-^13^C_4_]βOHB as described above. V_RaAcAc_ was calculated from V_RaβOHB_ turnover rates and the ratio of plasma βOHB to AcAc. V_CS_ was measured as described above. Hepatic acetate production rate (V_acetate_) was assumed to be negligible in humans.

### Quantification of hepatic RE production.

The production of REs, principally NADH and FADH_2_, can be estimated from the stoichiometry of the above fluxes as previously described (35, 72) using the equations:

(RE_AcAc_) = 3.5 × V_RaAcAc_

(RE_βOHB_) = 2.5 × V_RaβOHB_

(RE_βox-CS_) = 1.75 × V_CS_

(RE_TCA-only_) = 4 × V_CS_

(RE_GNG_) = V_glycerol-triose_ – (0.1 × V_PEP-triose_)

Total REs generated from β-oxidation were estimated as the sum of REs accounted for by ketogenesis (AcAc [RE_AcAc_] and βOHB [RE_βOHB_]) and TCA cycle turnover (RE_βox-CS_) as follows:

REs generated from β-oxidation (RE_βOX_) = RE_AcAc_ + RE_βOHB_ + RE_βox-CS_

Total RE turnover was calculated as the sum of REs from β-oxidation (RE_βOX_), TCA cycle dehydrogenases (RE_TCA-only_), and the dehydrogenases of GNG (RE_GNG_) using the equation:

 (Equation 10)
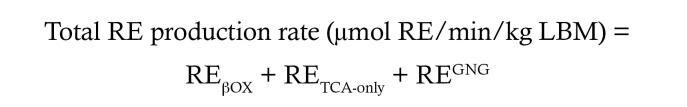


### Animal models and diets.

Hepatocyte-specific HMGCS2-null (HMGCS2-Liver-KO), BDH1-null (BDH1-Liver-KO), and *Hmgcs2* ASO–mediated null mice were generated as described previously (46, 48, 53, 55, 73, 74). Food and water were provided ad libitum. Mice were fed either a standard chow diet, a WD with 42% kcal from fat, an HFD with 60% kcal from fat, a choline-deficient methionine-limited HFD with 62% kcal from fat, or an HFCR diet with 90.5% kcal from fat.

### Mouse liver health assessment.

After euthanasia, mouse liver sections were frozen or fixed in 10% neutral-buffered formalin. Formalin-fixed tissue was used for histological analysis. Frozen liver tissue was assayed for total TAGs, glycogen content, oxidized lipid species, and abundance of mRNA markers.

### Serum NEFAs, TAGs, and blood glucose measurements.

Serum NEFAs (Wako, 633-52001) and TAGs (Thermo Fisher Scientific, TR22421) were measured spectrophotometrically using commercially available assays. Blood glucose was measured using glucose meters (Metene).

### Quantification of glucose, ketone bodies, and acetate.

Ketone bodies (AcAc and βOHB) were quantified using UHPLC-MS/MS as described previously (53, 56). Glucose, acetate, and βOHB were quantified during perfusions via ^1^H NMR.

### In vivo ^2^H/^13^C metabolic flux analysis in conscious unrestrained mice.

To measure hepatic metabolic fluxes in conscious mice, ^2^H_2_O, [6,6-^2^H_2_]glucose, and [U-^13^C]propionate were intravenously delivered into fasted mice, and then GC-MS was used to determine the isotopic enrichment of 3 glucose fragments. Using a metabolic reaction network constructed with Isotopomer Network Compartmental Analysis (INCA) software (http://mfa.vueinnovations.com/licensing/mfa-inca), which defines the carbon and hydrogen atom transitions for hepatic glucose and associated oxidative reactions, the relative reaction velocity (V) (i.e., flux) through each network reaction was determined relative to CS flux (V_CS_). The known [6,6-^2^H_2_]glucose infusion rate was then used to calculate absolute reaction velocities.

### Quantification of energy charge, redox state, and short-chain acyl-CoAs.

Energy nucleotides, redox nucleotides, and high-energy thioester containing acyl-CoAs were measured via LC-MS/MS, and energy charge and redox state were calculated (53).

### Flux calculations in perfused livers.

Stable isotope–based flux modeling used in vivo in humans and rodents as described above was used to study metabolism of the ex vivo–perfused liver as described previously, with minor modifications (52).

### Hepatic fat oxidation and RE production calculations in perfused livers.

In perfusions, total fat oxidation was calculated as:

 (Equation 11)
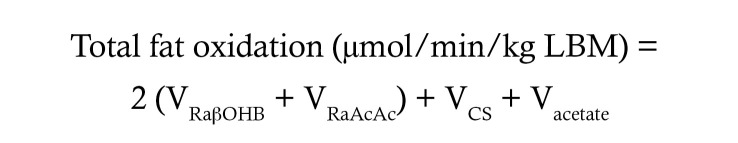


and expressed as μmol acetyl-CoA turned over/min/g liver. The same model, assumptions, and equations used to model RE production rate in vivo in humans were used to model RE production rate in ex vivo–perfused livers, with minor modifications.

### Mitochondrial respiratory control.

Liver mitochondria were isolated and steady-state oxygen consumption rates (*J*O_2_) were determined using a modified version of the creatine kinase (CK) energetic clamp as described previously (75). Mitochondrial NAD(P)H to NAD(P)^+^ ratio, acyl-CoAs and organic acids, and βOHB production were assayed after CK clamp.

### Statistics.

All analyses were performed using GraphPad Prism version 9. Unpaired 2-tailed Student’s *t* test or 1-way ANOVA was used to determine statistically significant differences. *P* less than 0.05 was accepted as significant for all tests. Information about statistical analysis is included in each figure legend. Data are presented as mean ± standard deviation (SD), unless otherwise indicated.

### Study approval.

The University of Minnesota’s Institutional Review Board approved the human study protocol and methods. All human participants provided written informed consent. All animal experiments were approved by the Institutional Animal Care and Use Committee at the University of Minnesota.

### Data availability.

All data required for evaluation of conclusions are included in this article. Individual data points are plotted in each graph or included in the Supporting Data Values file.

## Author contributions

Research studies were designed by EDQ, DBS, ABN, ABC, SBC, KF, DAD, JRR, ASW, DMM, SI, CCH, PP, and PAC. Data were acquired and analyzed by EDQ, DBS, ABN, ABC, SBC, KF, DAD, JRR, PJB, AH, JRG, SJ, FIR, ASW, DMM, CCH, and PP. SI and PAC also helped analyze data. Writing of the initial manuscript draft was done by EDQ, PP, and PAC. All authors edited the manuscript. Funding was acquired by SI, CCH, and PAC.

## Supplementary Material

Supplemental data

Supplemental tables 1-2

Supporting data values

## Figures and Tables

**Table 1:**
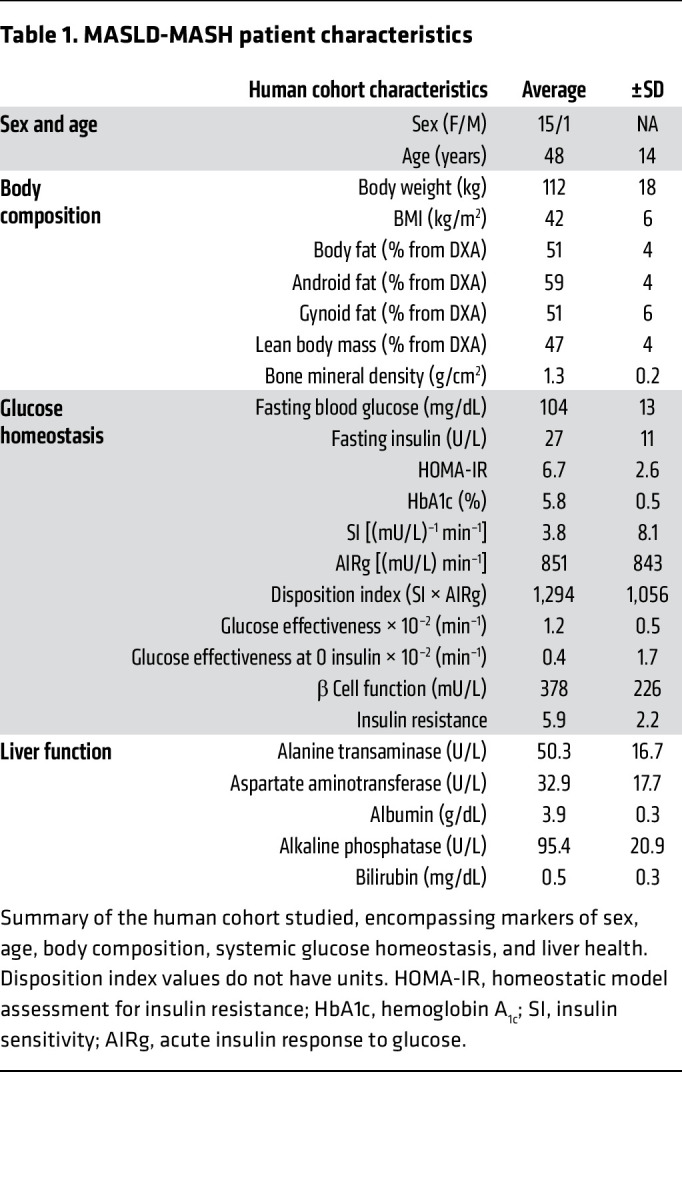
MASLD-MASH patient characteristics

## Version 1 04/24/2025

In-Press Preview

## Version 2 06/16/2025

Electronic publication

## Version 3 06/17/2025

Correction of figure 9B
