# Supplementary material for: Ketogenesis mitigates metabolic dysfunction–associated steatotic liver disease through mechanisms that extend beyond fat oxidation
Source: J Clin Invest. 2025 Apr 24;135(12):e191021. doi: 10.1172/JCI191021 (PMC12165805; doi:10.1172/JCI191021)

Supplemental Material

Ketogenesis mitigates metabolic dysfunction-associated steatotic liver disease through mechanisms that extend beyond fat oxidation

Eric D. Queathem[1,2,3], David B. Stagg[1], Alisa B. Nelson[1], Alec B. Chaves[4], Scott B. Crown[4], Kyle Fulghum[1], D. Andre d'Avignon[1], Justin R. Ryder[5,6], Patrick J. Bolan[7], Abdirahman Hayir[1], Jacob R. Gillingham[1,2], Shannon Jannatpour[8], Ferrol I. Rome[1], Ashley S. Williams[4], Deborah M. Muoio[4,9,10], Sayeed Ikramuddin[8], Curtis C. Hughey[1], Patrycja Puchalska[1*], Peter A. Crawford[1,2*]

[1]Division of Molecular Medicine, Department of Medicine, University of Minnesota Medical School, Minneapolis, MN, USA

[2]Department of Biochemistry, Molecular Biology and Biophysics, University of Minnesota Medical School, Minneapolis, MN, USA

[3]Department of Integrative Biology and Physiology, University of Minnesota Medical School, Minneapolis, MN, USA

[4]Duke Molecular Physiology Institute and Sarah W. Stedman Nutrition and Metabolism Center, Duke University Medical Center, Durham, NC, USA

[5]Department of Surgery, Lurie Children's Hospital, Chicago, IL, USA

[6]Department of Surgery and Pediatrics, Northwestern University Feinberg School of Medicine, Chicago, IL, USA

[7]Department of Radiology, University of Minnesota Medical School, Minneapolis, MN, USA

[8]Department of Surgery, University of Minnesota, Minneapolis, MN, USA

[9]Division of Endocrinology, Metabolism, and Nutrition, Department of Medicine, Duke University Medical Center, Durham, NC, USA

[10]Department of Pharmacology and Cancer Biology, Duke University Medical Center, Durham, NC, USA

*Contact for correspondence, crawforp@umn.edu or ppuchals@umn.edu

<u>Supplemental Methods</u>

*Human MASLD-MASH recruitment***.** Patients with abnormal liver function, prediabetes or type 2 diabetes were screened to identify participants who meet eligibility requirements for two clinical studies (ClinicalTrials.gov, NCT03997422 and NCT03587831). Participant data was de-identified for all data analysis. The full list of initial inclusion and exclusion criteria are provided in **Supplemental Table 1.** Patients enrolled were not given instruction or counseling to lose weight.

PDFF measurements. Each participant underwent a 20-minute MRI scan to measure liver fat content non-invasively. MRI scans were performed on a 3T Prisma MR system (Siemens Medical) at the UMN Center for Clinical Imaging Research. The PDFF image (MRI-PDFF) was acquired using a 3D GRE with 6 TE values (TR=9ms,flip angle=4, TE=1.15, 2.46, 3.69, 4.92, 6.15, 7.38 ms, 2x2x3.5mm resolution, one 17s breath-hold), and reconstructed using the vendor's software to produce a PDFF map.

Liver biopsy collection. Liver biopsies were collected by an interventional radiologist under direct image guidance with ultrasound-guided approach with percutaneous puncture using a 16-gauge Tru-cut needle and standard protocols in place to detect complications. Biopsies were scored independently by two blinded pathologists based on H&E-stained sections, assessing for macrovesicular steatosis (0-3), lobular inflammation (0-3), and acinar zone 3 hepatocellular injury, or ballooning degeneration (0-2), which were summed to express the NAFLD activity score, NAS. Fibrosis (0-4) was also quantified using picrosirius red staining.

Assessment of glucose homeostasis via FSIVGTT. For three days prior to admission to the clinical research unit (CRU), participants consumed a weight-maintaining diet formulated to approximate the previous 3-day dietary record. An evening meal consisting of 40% daily caloric intake and 40-50% carbohydrate and 30-40% fat was consumed before the 0700 CRU admission for FSIVGTT. Two intravenous catheters were placed, one for sampling and the other for administration of dextrose and insulin. Glucose, insulin and C-peptide were quantified at baseline and multiple times over the course of 180 minutes. HOMA-IR was calculated using the equation, HOMA-IR = fasting insulin (mU/L) × fasting glucose (mg/dL) / 405. At time 0, dextrose 0.3 grams/kg BW was given intravenously. Insulin, 0.03 units/kg BW was then injected at 20 minutes to augment glucose disposal. Insulin and C-peptide levels were measured for 10 minutes and the area under the curve was calculated as a measure of insulin secretory function, termed AIRg. A minimal model assessment was done to calculate total body insulin resistance (Si), then the Disposition Index (DI) was calculated as the product of AIRg x Si. For FSIVGTT modeling, glucose was measured via UV test enzymatic reference method with hexokinase, insulin was measured via electrochemiluminescence immunoassay (ECLIA), and C-peptide was measured via chemiluminescent immunoassay.

Body composition measurements via DXA imaging. Body composition was measured by a DXA scanner, the GE Healthcare Lunar i (GE Healthcare Lunar with enCORE software version 16.2).

Stable isotope delivery in humans. After FSIVGTT and DXA scan, participants remained in the CRU and received a low-carbohydrate lunch meal. Participants then began

fasting and did not receive an evening meal. Stable isotope intake began at 2200 hours. Participants were provided with deuterated water (^2H_2O, D_2O, 5 g/kg body water, calculated as 60% of BW in men and 50% of BW in women) (CIL, DLM-2259-70-1L) every 4 hours between 2200 and 0600. These doses, along with 0.5% ^2H_2O available for ad libitum consumption, sufficiently labeled body water for measuring flux parameters. From 0800 to 0900, [U-$^{13}C_3$]propionate (CIL, CLM-1865-MPT-PK) was administered orally as two 300 mg doses. At 0900, intravenous primed continuous stable isotope infusion of [3,4-$^{13}C_2$]glucose (Omicron, GLC-061; or CIL, CLM-6750-MPT-PK) and D-[U-$^{13}C_4$]βOHB (CIL, CLM-3853-PK) began with samples collected at metabolic and isotopic steady-state (~2hr after start of infusion).

^2H/^{13}C NMR data acquisition. MAG was reconstituted in 510 µL acetonitrile spiked with 5% ^2H-depeleted H_2O, then was transferred to a 5 mm 528-pp Wilmad NMR tube and analyzed on a Bruker Avance III 600 MHz NMR instrument equipped with a CryoProbe. During ^2H NMR data collection, the magnet was not maintained on a field-frequency lock, as the lock channel is used to acquire ^2H signal. Data were collected at 25°C over 1380 Hz using a 68 µs (90°) pulse, 1.3s delay between transients and Waltz16 decoupling on the proton channel during a 1s acquisition period. Depending on signal-to-noise (S/N) 4,000-40,000 transients were collected. Magnet stability was sufficient to result in deuterium line-widths of 2-3 Hz, allowing for integration to establish relative ^2H enrichments at the number 2, 5 and 6 positions of ^2H-labeled MAG, reflecting the relative ^2H enrichments of plasma glucose. For ^{13}C NMR data collection, 40 µL of d_3-acetonitrile was spiked into each sample used to collect the ^2H NMR spectra, then a field-frequency lock was established. For data acquisition, a 12 µs excitation pulse with

22,320 Hz collection window and waltz16 decoupling for ^{1}H was used. Depending on S/N 4,000 to 40,000 transients were collected then signal magnitudes were established by integration. The ^{13}C isotopomers that appear in glucose provide a readout on oxidative metabolism in the liver, which are encoded in the multiplet pattern arising at the C2 resonance of MAG, centered at ~85 ppm. The relative populations of the D12 doublet, D23 doublet and Q123 quartet that arise when carbons 1 and 2, carbons 2 and 3, or carbons 1, 2 and 3 are labeled, respectively, then were used to mathematically model TCA cycle turnover, anaplerosis, cataplerosis, and pyruvate cycling as described below.

Quantifying plasma glucose fractional ^{13}C enrichment. The fractional enrichment of plasma glucose was determined from the ^{13}C NMR spectra of MAG by measuring the ratio (R) of enrichment at C3 and C4, relative to the natural abundance of the methyl groups from the acetone moiety of the MAG derivative. Fractional ^{13}C enrichment (L_p) of the plasma glucose pool was then calculated using linear regression, from a standard curve of known glucose enrichments and the equation $L_p = 1.33\ (R) + 0.031$.

Quantifying hepatic oxidative fluxes. At metabolic steady-state, glucose-linked rates of anaplerosis must equal cataplerosis, which are modeled relative to the turnover rate of the hepatic TCA cycle, assumed to be equal to the rate of CS (V_{CS}). Because the non-oxidative entry (anaplerosis) and exit (cataplerosis) of carbon through the TCA cycle supports glucose anabolism via PEP-sourced GNG, changes in oxidative fluxes distort the ^{13}C enrichment patterns of PEP which are transmitted and encoded in the multiplet resonances of plasma glucose. Using the known rate of PEP-derived GNG (V_{PEP}), quantified as described above, and converted into triose units by multiplying by 2, and

the ^{13}C isotopomer populations quantified via ^{13}C NMR, the absolute rate of the TCA cycle turnover (V_{CS}), as well as anaplerotic (V_{PC}) and cataplerotic fluxes (V_{PEPCK}), and label recycling rates (V_{PK+ME}) can be calculated. Pyruvate cycling (V_{PK+ME}) reflects the summed rate of pyruvate kinase (PK) and malic enzyme (ME). Total anaplerosis is modeled as flux of PC (V_{PC}), while cataplerosis is modeled as flux through PEP carboxykinase (PEPCK) (V_{PEPCK}), which at steady-state are equal ($V_{PC} = V_{PEPCK}$).

Quantifying hepatic RE production. To calculate the production rate of REs, principally NADH and $FADH_2$, this approach assumes that all acetyl-CoA originates from palmitate and that the sourcing of V_{PEP} reflects 90% lactate and 10% pyruvate/alanine, in accordance with the typical hepatocellular redox state. The model assumes that complete β-oxidation of palmitate, a 16-carbon saturated FA, yields a total of eight acetyl-CoA units, requiring seven rounds of the β-oxidation cascade which collectively yields 14 REs, or an average of 1.75 REs per molecule of acetyl-CoA produced (14 REs / 8 Ac-CoA = 1.75 REs / Ac-CoA). By quantifying the rate for each disposal pathway for acetyl-CoA, the turnover of REs generated from fat oxidation can be calculated from the stoichiometry of each pathway as follows. For every mole of AcAc produced, two moles of acetyl-CoA turned over, and thus total REs generated from β-oxidation that are accounted for by AcAc **(RE_{AcAc})** = 3.5 x V_{RaAcAc}. For every mole of βOHB produced, two moles of acetyl-CoA turned over, but conversion of AcAc to βOHB consumes 1 RE, and thus net REs generated from β-oxidation that are accounted for by βOHB **($RE_{\beta OHB}$)** = 2.5 x $V_{Ra\beta OHB}$. For every turn of the TCA cycle, one molecule of acetyl-CoA is consumed by CS, accounting for 1.75 REs, but each turn also generates an additional 4 REs, and thus total REs accounted for by the TCA cycle **($RE_{TCA\text{-}total}$)** = 5.75 x V_{CS}. $RE_{TCA\text{-}total}$ can

be broken down into (1) total RE's generated from β-oxidation **($RE_{\beta ox\text{-}CS}$)** = 1.75 x V_{CS}, and (2) total REs generated exclusively from the dehydrogenases of the TCA cycle (not β-oxidation) **($RE_{TCA\text{-}only}$)** = 4 x V_{CS}. Total REs generated from β-oxidation can then be estimated as the sum of REs accounted for by AcAc, βOHB, and the TCA cycle dehydrogenases as follows:

REs generated from β-oxidation **($RE_{\beta OX}$)** = $RE_{AcAc} + RE_{\beta OHB} + RE_{\beta ox\text{-}CS}$

Total RE balance in the liver requires further accounting for GNG and three enzymes in particular: glycerol-3-phosphate dehydrogenase (G3PDH), glyceraldehyde-3-phosphate dehydrogenase (GAPDH), and lactate dehydrogenase (LDH). G3PDH generates 1 RE during GNG from glycerol. GAPDH consumes 1 RE during GNG from PEP, however, if the substrate used to source PEP is lactate, then LDH will generate 1 RE to offset this. Assuming that GNG is sourced 90% from lactate, and 10% from pyruvate + alanine, and expressing $V_{Glycerol}$ and V_{PEP} as triose units (by multiplying by 2), then total REs generated from GNG **(RE_{GNG})** = $V_{Glycerol\text{-}Triose}$ – (0.1 x $V_{PEP\text{-}Triose}$). From this, total REs turned over in the liver can then be calculated at steady-state as the sum of REs generated from β-oxidation, TCA cycle flux, and GNG:

Total RE production rate (μmol RE/min/kg-LBM) = $RE_{\beta OX} + RE_{TCA\text{-}only} + RE_{GNG}$

Animal models and diets. All mice were bred on a C57BL/6NJ background and received autoclaved water ad libitum. Mice were housed on corncob bedding in groups of 4 to 5 with lights off between 2000 and 0600 in a room maintained at 22°C. Hepatocyte-specific HMGCS2 null (HMGCS2-Liver-KO) mice were generated as described previously by crossing homozygous *Hmgcs2*$^{flox/flox}$ mice, with heterozygous Cre

recombinase expressing mice, driven by the albumin promoter (1–3). Littermate wild-type (WT) Alb-Cre-negative (*Hmgcs2*$^{flox/flox}$, Alb-Cre$^{+/+}$) animals were used as controls for Cre-positive (*Hmgcs2*$^{flox/flox}$, Alb-Cre$^{+/Tg}$) HMGCS2 KO mice, which are maintained on a C57BL/6NJ substrain hybrid background. Hepatocyte-specific BDH1 null (BDH1-Liver-KO) mice were generated as previously described (4), by crossing homozygous *Bdh1*$^{flox/flox}$ mice (5) to heterozygous Cre recombinase expressing mice, driven by the albumin promoter (2). Littermate WT Cre-negative (*Bdh1*$^{flox/flox}$, Cre$^{+/+}$) animals were used as controls for Cre-positive (*Bdh1*$^{flox/flox}$, Cre$^{+/Tg}$) BDH1-Liver-KO mice. *Hmgcs2* ASO-mediated loss of function was initiated in 6-week-old mice by i.p. injection (25 mg/kg) with murine *Hmgcs2*-targeted ASOs or scrambled sequence control ASOs (Ionis Pharmaceuticals) biweekly as described previously (6). Standard chow diet (Teklad Global, 2016) consisted of 12% kcal from fat, 66% kcal from carbohydrates and 22% kcal from protein. Western diet (Envigo, TD.88137) consisted of 42% kcal from fat, 43% kcal from carbohydrates and 15% kcal from protein. 60% high-fat diet (Research Diets, D12492) consisted of 60% kcal from fat, 20% kcal from carbohydrates, and 20% kcal from protein. HFCR diet (Envigo, TD.160153.PWD) consisted of 90.5% kcal from fat, 0% kcal from carbohydrates and 9.5% kcal from protein. The choline-deficient, methionine-limited high fat diet (Research Diets, A06071302) consisted of 62% kcal from fat, 21% kcal from carbohydrates and 18% kcal from protein.

Mouse liver histology. For histology, tissue embedding, sectioning, and staining with hematoxylin and eosin, and picrosirus red, was performed by the University of Minnesota Clinical and Translational Science Institute histology core.

Quantifying liver TAGs. 20 mg frozen liver tissue was homogenized in 1 mL of 0.1x PBS, then total protein was quantified via BCA assay. Total lipids were extracted from 0.5 mg of protein via a Bligh-dyer lipid extraction, then total lipids were quantified colorimetrically using Infinity TAG reagent (Thermo, TR22421).

Quantifying liver glycogen content. Total glycogen was assayed as described previously (7). Briefly, 100 mg of frozen liver tissue was digested in 300 µL of 30% KOH (w/v), then glycogen was precipitated followed by enzymatic digestion using 0.3 mg/mL amyloglucosidase in 0.2 M sodium acetate buffer at pH 4.8 (Sigma 10115, 70 U/mg). Total glucose was quantified using ^{1}H NMR then total glycogen was expressed in glucose units per mg of liver tissue.

Quantification of hydroxyalkenal species in liver tissue. Frozen liver tissue was homogenized in ice-cold water using 2.8- and 1.4-mm zirconium oxide beads at 4°C, then an appropriate amount of internal standard was spike in based on total protein determined using the BCA assay, followed by lipid extraction using a modified Bligh-dyer as previously described (3, 7). Lipid extracts were then derivatized with carnosine and quantified using shotgun lipidomics as previously described (8).

Quantification of total ketone bodies via UHPLC-MS/MS. Briefly, [U-$^{13}C_4$]AcAc and [3,4,4,4-D_4]βOHB internal standards were spiked into ice cold MeOH:ACN (1:1), then ketones were extracted from serum, perfusate, plasma, or sample extracts, followed by separation via reverse-phase UHPLC, and detected via parallel reaction monitoring (PRM) on a QExactive Plus hybrid quadrupole-orbitrap mass spectrometer. Quantification of ^{13}C-labeled ketone bodies was achieved post reduction with $NaBD_4$ as previously described (3, 9).

Quantification of glucose, βOHB, and acetate during liver perfusions via ^1H NMR. 1.8 mL of liver effluent were collected, snap frozen and stored at -80°C till analysis. To prepare samples, the solvent was removed at 45°C under vacuum, then samples were reconstituted in 550 µL of ^2H_2O (99.9%) spiked with 0.3 mM [d_8]-trimethyl-silyl propionate (TSP). ^{1}H-NMR signals were acquired using a Bruker Avance III 600 MHz NMR instrument equipped with a CryoProbe, then the integrated intensities of the α-anomeric proton on glucose carbon-1, the methyl signal for βOHB, the methyl signal for acetate, and the tri-methyl signal from TSP, were used to calculate molar concentrations of the respective substrates. For all ^{1}H-NMR collections, spectra were collected by conventional pulse-and-collect measurements under quantitative conditions (10-ppm spectral range using ~15 µs [90°] excitation pulse and 22-second delay between each of 20 transients).

Stable isotope infusions in mice. Jugular vein and carotid artery catheters were implanted into mice for isotope infusion and sampling as previously described (10–12). The exteriorized ends of the implanted catheters were flushed with 200 U·mL^{-1} heparinized saline and sealed with stainless-steel plugs. After surgery, mice were housed individually for ~7 days of postoperative recovery before stable isotope infusions studies. All mice were within 10% of presurgical weight before stable isotope infusions. Mice were fasted during the duration of isotope infusions, and were fasted a total of 7 hours when samples were collected. Two hours into the fast, the exteriorized catheters were connection to infusion syringes. After a 1-hour acclimation period, an arterial blood sample (80 µL) was collected to determine glucose natural abundance enrichment, followed by a stable isotope infusion protocol to measure glucose-linked hepatic oxidative fluxes as previously described (12, 13). Briefly, a ^2H_2O (99.9%)-saline

bolus (CIL, DLM-4-PK) containing [6,6-$^2H_2$]glucose (99%) (CIL, DLM-349-MPT-PK) was infused over 25 min to enrich body water and prime (440 μmol·kg^{-1}) the glucose pool. This was followed by a continuous infusion of [6,6-$^2H_2$]glucose (4.4 μmol·kg^{-1}·min^{-1}) till the end of the experiment. A primed (1.1 mmol·kg^{-1}), continuous (0.055 mmol·kg^{-1}·min^{-1}) intravenous infusion of [U-^{13}C]propionate (CIL, CLM-1865-MPT-PK) was started 2h after the ^2H_2O bolus. 100 μL arterial samples were collected at 90, 100, 110, and 120 mins after the [U-$^{13}C_3$]propionate bolus to determine arterial glucose concentration and enrichment. Donor erythrocytes were provided by constant rate infusion throughout the experiment to prevent a decline in hematocrit. Mice were euthanized by cervical dislocation following the final sample collection, then tissues were rapidly excised, freeze-clamped in liquid nitrogen. All samples were stored at −80°C.

Glucose derivatization, GC-MS analysis and in vivo *^{2}H/^{13}C metabolic flux analysis in conscious unrestrained mice.* Approximately 40 μL of plasma at each time point was used to synthesize di-*O*-isopropylidene propionate, aldonitrile pentapropionate, and methyloxime pentapropionate derivatives of glucose, then GC-MS analysis was utilized to collect uncorrected mass isotopomer distributions (MIDs) for six fragment ions as previously described (13–15). The metabolic flux analysis methodology used in these studies followed that previously described with minor modifications (14). To summarize, a metabolic reaction network was constructed using Isotopomer Network Compartmental Analysis (INCA) software (16). The reaction network defined the carbon and hydrogen atom transitions for hepatic glucose and associated oxidative reactions. The reaction velocities (i.e., flux) through each network reaction was determined relative

to CS flux (V_{CS}) by minimizing the sum of squared residuals between simulated and experimentally determined MIDs of the six fragment ions described above. Flux estimates were repeated 50 times from random initial values. Goodness of fit was assessed by a Chi-square test (P = 0.05) and confidence intervals of 95% were determined as previously described (12). Mouse body weights and the [6,6-$^2H_2$]glucose infusion rate were used to determine absolute values. In the GCMS-based model, total EGP (V_{EGP}) is equal to the sum of glycogenolysis (V_{PYGL}) and GNG (V_{Aldo}), which are modeled as the rates of glycogen phosphorylase (PYGL) and aldolase (Aldo), respectively. Sourcing of GNG from glycerol (V_{GK}) and PEP (V_{Enol}) are modeled as the rates of glycerol kinase (GK) and enolase (Enol), respectively. Pyruvate cycling (V_{PK+ME}) is equal to the summed rate of PK and ME and total cataplerosis is still equal to flux through PEPCK (V_{PCK}). Anaplerosis is equal to the sum of PC (V_{PC}) and propionyl-CoA carboxylase (PCC) (V_{PCC}), and accounts for entry of unlabeled substrates through LDH (V_{LDH}). Turnover of the 1st span of the TCA cycle is modeled as the rate of CS (V_{CS}), whereas turnover of the 2nd span of the TCA turnover is modeled as the rate of succinate dehydrogenase (SDH) (V_{SDH}), and reflects the sum of V_{CS} and V_{PCC}.

Quantifying energy charge, redox state and short-chain acyl-CoAs. Energy nucleotides (ATP, ADP, AMP), redox nucleotides (NADH, NAD^+), and high-energy thioester containing acyl-CoAs (acetyl-, succinyl-, propionyl-CoA) were extracted, separated using ion-pairing reverse-phase UHPLC-MS/MS on a C_{18} column (Waters Xbridge, 150 x 2.1mm, 3µm), then detected on a Thermo QExactive Plus mass spectrometer, operated in positive ionization mode, using PRM transitions as previously described (4, 17). Relative pool size (ion counts) normalized to biomass (mg total protein) was

quantified for all analytes. Nucleotides were detected as adducts of dibutylammonium acetate. Energy charge and Redox State was then estimated in the liver using the pool size of nucleotides and the equations:

$$\text{Energy Charge} = \frac{([\text{ATP}]+0.5[\text{ADP}])}{\text{Total Energy Nucletodies}}$$

$$\text{Total Energy Nucleotides} = [\text{ATP}] + [\text{ADP}] + [\text{AMP}]$$

$$\text{Redox State} = \frac{[\text{NAD+}]}{[\text{NADH}]}$$

$$\text{Total Redox Nucleotides} = [\text{NAD} +] + [\text{NADH}]$$

Ex vivo mouse liver perfusions. Liver perfusions were performed as described previously (7). Briefly, mice were anesthetized with i.p. sodium pentobarbital injection, externally sterilized with 70% ethanol, then mice were dissected to expose the hepatic portal vein, followed by cannulation with a 24-gauge catheter needle. The cannula was secured, then the abdominal aorta, inferior vena cava and cardiac right atrium were severed to isolate the liver from peripheral tissues and prevent recirculation. Livers were perfused with oxygenated Krebs-Henseleit buffer (118 mM NaCl, 4.7 mM KCl, 2.5 mM $CaCl_2$, 1.2 mM KH_2PO_4, 1.22 mM $MgSO_4$, 25 mM $NaHCO_3$, pH 7.4), warmed to ~45^oC and supplemented with 1.5 mM sodium lactate, 0.15 mM sodium pyruvate, 0.25 mM glycerol, 0.1 mM [U-$^{13}C_3$]propionate and 3% ^2H_2O. Perfusion buffers were also supplemented with exogenous FAs. Either 0.2 mM sodium octanoate was added directly to buffers, or 0.8 mM of a LCFA mixture (30% palmitate, 35% oleate, 8% stearate, and 27% linoleate) was bound to FA-free BSA at a BSA:FA molar ratio of 1:3, then dialyzed using a capillary dialysis filtration system and a Reveclear-300 dialysis

cartridge (Baxter, 114745L) prior to addition to buffer. Specifics of buffer composition can be found in each figure legend. Perfusion buffer was oxygenated by continuous bubbling of a 95% O_2, 5% CO_2 gas mixture through the buffer reservoir using a fritted glass tube, and was delivered using a peristaltic pump at a flow rate of ~8 mL/min for 60 minutes. Buffer and effluent samples were collected every 10 minutes. At the completion of the perfusion, the liver was freeze-clamped in liquid nitrogen, and subsequently weighed to obtain total liver weight for flux normalizations. Perfusion buffer (input), effluent (output) and tissues were stored at -80°C until analysis.

Flux calculations in perfused livers. Briefly, total EGP rates **(V_{EGP})** was calculated from the rate of appearance of unlabeled glucose in the perfusate. βOHB production **($V_{Ra\beta OHB}$)** and AcAc production **(V_{RaAcAc})** was calculated from the rate of appearance of unlabeled ketones in the perfusate. Acetate production **($V_{Acetate}$)** was quantified as the appearance of unlabeled acetate in the perfusate, and included in the ex vivo LCFA oxidation measurements. For octanoate perfused livers, it was assumed that very little acetate arose from octanoate, and therefore was not included in these calculations. Livers were perfused with $^{2}H_2O$ and [U-$^{13}C_3$]propionate, which generate positional isotopomer populations that allow intrahepatic fluxes to be quantified as described during in vivo experiments in humans and mice. The ^{2}H-enrichment of glucose at H2, H5 and H6s reports on the fractional contribution of glycogen, glycerol and PEP to glucose production. V_{EGP} measured from total glucose production in the perfusate, therein allows $V_{Glycogen}$, $V_{Glycerol}$ and V_{PEP} to be quantified as described above. The ^{13}C-enrichment of glucose at carbons C1, C2 and C3 from [U-$^{13}C_3$]propionate distort the multiplet nuclear magnetic resonances of the C2 carbon in a manner that quantitatively

encodes the ratio of TCA cycle turnover relative to anaplerosis, cataplerosis and pyruvate cycling. Because at steady-state the oxidative metabolic network herein described is coupled to PEP-linked GNG, by normalizing fluxes to V_{PEP} (measured as described above), absolute fluxes through the TCA cycle, anaplerosis, cataplerosis and pyruvate cycling can be calculated.

RE production calculations in liver perfusions. For liver perfusions, REs from each pathway were calculated using the same equations as in humans (described above). For LCFA liver perfusions only, a $RE_{Acetate}$ term ($RE_{Acetate} = 1.75 \times V_{Acetate}$) was included. For octanoate perfused livers, the following modifications were made. Because octanoate only generates four acetyl-CoA molecules, via three rounds of β-oxidation which generate six REs, the ratio of REs generated per acetyl-CoA molecule is 1.5 (6 RE / 4 Ac-CoA = 1.5), and not 1.75 as when palmitate is metabolized. Based on this the REs generated from each pathway can be calculated as follows:

$$RE_{AcAc} = 3 \times V_{RaAcAc}$$

$$RE_{\beta OHB} = 2 \times V_{Ra\beta OHB}$$

$$RE_{TCA\text{-}total} = 5.5 \times V_{CS}$$

$$RE_{TCA\text{-}only} = 4 \times V_{CS}$$

$$RE_{\beta ox\text{-}TCA} = 1.5 \times V_{CS}$$

$$RE_{\beta OX} = RE_{Acetate} + RE_{AcAc} + RE_{\beta OHB} + RE_{\beta ox\text{-}TCA}$$

Total RE production rate can then be calculated based on $V_{Glycerol}$ and V_{PEP}, expressed as triose units, based on the assumptions that V_{PEP} is sourced 90% from lactate and 10% for pyruvate/alanine.

$$RE_{GNG} = V_{Glycerol\text{-}Triose} - (0.1 \times V_{PEP\text{-}Triose})$$

$$\text{Total RE produced (μmol/min/g liver)} = RE_{\beta OX} + RE_{TCA\text{-}only} + RE_{GNG}$$

Liver mitochondrial isolation. Mice were fasted for 18h with access to water. Livers were excised and placed in ice-cold mannitol-sucrose isolation media (MSIM; 220 mM Mannitol, 70 mM sucrose, 10 mM Tris, 1mM EDTA; pH=7.2) supplemented with 5 g/L fatty-acid free bovine serum albumin (BSA) (MSIM+BSA). Livers were minced in 5 mL MSIM and liver pieces were rinsed three times in MSIM+BSA then transferred to an ice-chilled borosilicate glass vessel for homogenization. Homogenized liver tissue was centrifuged at 700 x g for 10 min at 4°C and the supernatant was filtered through two layers of gauze and centrifuged at 9,000 x g for 10 min at 4°C. The supernatant was discarded, and pellets were gently resuspended in 10 mL MSIM+BSA and centrifuged at 7,000 x g for 5 min at 4°C. The supernatant was discarded, and pellets were resuspended in 1.2 mL MSIM+BSA and centrifuged at 9,000 x g at 4°C. MSIM+BSA was aspirated from each tube and mitochondrial pellets were resuspended in 1.2 mL of MSIM without BSA and centrifuged at 9,000 x g at 4°C. MSIM without BSA was aspirated from each tube and mitochondrial pellets were resuspended in MSIM without BSA. Protein content was determined via a BCA protein assay and mitochondria were resuspended at a final concentration of 20 mg/mL for all functional assays.

Mitochondrial respiratory control. Briefly, isolated mitochondria (0.150 mg/mL) were added to Buffer Z (105 mM MES potassium salt, 30 mM KCl, 10 mM KH_2PO_4, 5 mM $MgCl_2$) supplemented with Creatine (5 mM), PCr (1.5 mM), CK (20 U/mL), fatty acid free BSA (2.5 mg/mL), and EGTA (1 mM) with either 10 μM Hymeglusin (HG) or the vehicle control (DMSO), followed by the addition of respiratory substrates (Pc/AKG; 50 μM Palmitoyl-L-Carnitine, 5 mM alpha-ketoglutarate), and ATP (5 mM). Next, sequential additions of PCr was used to adjust the free energy of ATP hydrolysis (ΔG_{ATP}). Following the respiration assays, 1.6 mL of the mitochondrial buffer slurry was immediately collected and chilled on ice-water prior to differential centrifugation (9,000 x *g* for 5 minutes at 4 °C) to separate the mitochondrial supernatant. Supernatants were flash frozen on dry-ice and stored at -80°C until further analysis.

Mitochondrial incubations for metabolite profiling. Isolated liver mitochondria (0.2mg/mL) were incubated in 2 mL Buffer Z supplemented with creatine (Cr; 5 mM), phosphocreatine (PCr; 1.5 mM), creatine kinase (CK; 20 U/mL), sodium bicarbonate ($Na^+HCO_3^-$; 2 mM), respiratory substrates (Pc/AKG), and either 10 μM Hymeglusin (HG) or the vehicle control (VCTRL; DMSO), for 5 minutes at 37°C with constant stirring. Subsequently, 5 mM of ATP was added to reach respiratory state 3, followed by a 5-minute incubation at a fixed energy demand (ΔG_{ATP} = -13.94 kcal/mol) imposed by the addition of PCr (final concentration = 6 mM). 1.6 mL of the mitochondrial buffer slurry was immediately collected and chilled on ice-water prior to differential centrifugation (9,000 x *g* for 5 minutes at 4 °C) to separate the mitochondrial pellet and supernatant. Both fractions were flash frozen on dry-ice and stored at -80°C until further analysis.

Measuring mitochondrial βOHB production. Following the mitochondrial respiration control assays described above, 1.6 mL of the mitochondria-buffer slurry was immediately collected and chilled on ice-water prior to differential centrifugation (9,000 x *g* for 5 minutes at 4°C) to isolate the mitochondrial supernatant. βOHB levels in the supernatant were determined via a commercial kit (Wako Autokit 3-HB, Fujifilm Healthcare Solutions), according to manufacturer specifications, with modifications. Briefly, the principle of the assay is to colorimetrically monitor the reduction of $NAD^+ \rightarrow NADH$ coupled to the oxidization of βOHB present in the assay samples. The reaction is catalyzed by the addition of βOHB dehydrogenase present in the R2 working reagent. To ensure AcAc does not interfere with the absorbance readings, AcAc in the samples is converted to acetone following the addition of acetoacetate decarboxylase present in the R1 working reagent. Prior to running the assay, mitochondrial supernatants were diluted 3-fold in Buffer Z. 20 µL of mitochondrial supernatant samples and βOHB standard curve samples (0-12.5 µM) were loaded onto a 96-well flat-bottom plate in duplicate. 180 µL of R1 working reagent was added to the standard and sample wells to monitor background absorbance at 405 nm for 5 minutes 37°C. Following, 60 µL/well of R2 working reagent was added, and absorbance was kinetically monitored 405 nm was monitored for 24 minutes at 37°C. For each of the βOHB standards, absorbance slope values (Δ absorbance/Δ seconds) were used to derive a linear standard curve to derive βOHB concentration present in each unknown sample. Unknown values were corrected for protein and total assay volume and expressed as nmol βOHB/mg protein.

Measuring mitochondrial NAD(P)H/NAD(P)+ redox. Fluorescent measurements of mitochondrial $NAD(P)H/NAD(P)^+$ redox were determined using a QuantaMaster

Spectrofluorometer (QM-400; Horiba Scientific) as described previously (18). All assays were conducted at 37°C in a 0.2 mL reaction buffer. The buffer for all assays was Buffer Z supplemented with creatine (Cr; 5 mM), phosphocreatine (PCr; 1.5 mM), creatine kinase (CK; 20 U/mL), and either 10 μM Hymeglusin (HG) or DMSO (VCTRL). At the start of each assay, isolated liver mitochondria (0.2 mg/mL) were added to the assay buffer, followed by the addition of respiratory substrates (Pc/AKG) alone to capture 100% reduction within the $NAD(P)H/NAD(P)^+$ couple, and then sequential additions of PCr to obtain the following final concentrations: 3, 6, 9, 12, 15 mM. After the final PCr addition, alamethicin (12.5 μg/mL) was added to permeabilize the mitochondria. $NAD(P)H/NAD(P)^+$ during the experiment was expressed as a percent reduced according to the formula: % Reduction = (F-F0%)/(F100%-F0%), where the 0% reduction state is represented as the fluorescent signal (Ex/Em, 340/450nm) recorded in the presence of alamethicin.

Mitochondrial acyl-CoA analysis by LC-MS/MS. Acyl-CoA analysis was performed as described in previous reports (18–20). Briefly, frozen mitochondrial pellets (0.4 mg) were sonicated for ~5 minutes in 1.1 mL extraction buffer (1:1 methanol/water containing 5% acetic acid) containing 20 μL 0.01 mM [D_9]-pentanoyl-CoA as internal standard. Homogenates were centrifuged at 4°C for 15-minute at 18,000 x g and 900 μL of supernatant was used for solid phase extraction (SPE). For SPE, the cartridge (1 mL ion exchange cartridge packed with 100 mg of 2-2(pyridyl)ethyl silica gel (Sigma)) was pre-activated with 1 mL of methanol and then with 1 mL of extraction buffer (1:1 methanol/water containing 5% acetic acid). The acyl-CoAs trapped on the silica gel cartridge were released with (1) 1 mL of a 1:1 mixture of 50 mM ammonium formate (pH

6.3) and methanol; (2) 1 mL of a 1:3 mixture of 50 mM ammonium formate (pH 6.3) and methanol; and (3) 1 mL of methanol. The combined effluent was dried with nitrogen gas and stored at −80°C until LC-MS analysis. A 40 μL sample was injected on a Pursuit XRs 5 C_{18} column (150 × 2.0 mm, 5μm), protected by a guard column (Pursuit XRs 5 C_{18} 5 μm, 10 × 2.0 mm, 5μm) with temperature controlled at 25°C, in an ExionLC AD liquid chromatograph system. The Analyst software (version 1.6) was used for data record and processing. Relative pool size estimates were determined by comparing analyte peak areas to [D_9]-pentanoyl-CoA peak area, then normalized to the VCTRL (set to 1) to represent fold-change in the pool size of CoA in the presence of HG. The detailed LC-MS/MS conditions can refer to our previous reports (18–20).

Mitochondrial organic acid profiling by GC-MS. Organic acids in the incubation buffer were methoximated and silylated to TBDMS-derivatives and profiled by a gas chromatography mass spectrometry (GCMS) method. Briefly, 200 μL of incubation buffer was spiked with 20 μL 0.05 mM norvaline as internal standard and then vortexed vigorously with 200 μL methanol and 1 mL acetonitrile. Homogenates were then centrifuged at 4°C for 15-minute at 18,000 x g and 675 μL of the resulting supernatant was dried under nitrogen gas at 37°C. TBDMS-derivatives were prepared for GC-MS with MOX and TBDMS as described previously (21, 22). GC-MS analyses were performed on an Agilent 7890B GC system equipped with a HP-5MS capillary column (30 m, 0.25 mm i.d., 0.25 μm-phase thickness; Agilent J&W Scientific), connected to an Agilent 5977A Mass Spectrometer operating under ionization by electron impact (EI) at 70 eV. Relative pool size estimates were determined by comparing analyte peak areas to norvaline peak area (m/z 288).

Gene expression. For mRNA expression analysis, samples were harvested and homogenized in Qiazol, then total RNA was isolated using Qiagen's RNeasy protocol. First-strand cDNA was synthesized using iScript (BioRad, 170-8891), then quantitative real-time PCR was performed on a BioRad CFX384 Real-Time thermocycler using SsoAdvanced Universal SYBR Green supermix (BioRad, 170-5274). mRNA expression was calculated using the $2^{-\Delta\Delta Ct}$ method, where ΔCt = housekeeping gene Ct – gene of interest CT, then expressed as fold-change relative to the intact liver. mRNA levels were normalized to the intact liver, which was set to 1. Housekeeping gene was *Rpl32* for all targets. Primer sequences are given in **Supplemental Table 2.**

References

1. Alexander M, et al. A diet-dependent host metabolite shapes the gut microbiota to protect from autoimmunity. *Cell Rep*. 2024;43(11). https://doi.org/10.1016/j.celrep.2024.114891.

2. Postic C, et al. Dual Roles for Glucokinase in Glucose Homeostasis as Determined by Liver and Pancreatic β Cell-specific Gene Knock-outs Using Cre Recombinase*. *J Biol Chem*. 1999;274(1):305–315.

3. Queathem ED, et al. Ketogenesis supports hepatic polyunsaturated fatty acid homeostasis via fatty acid elongation. *Sci Adv*. 2025;11(5):eads0535.

4. Stagg DB, et al. Diminished ketone interconversion, hepatic TCA cycle flux, and glucose production in D-β-hydroxybutyrate dehydrogenase hepatocyte-deficient mice. *Mol Metab*. 2021;53:101269.

5. Horton JL, et al. The failing heart utilizes 3-hydroxybutyrate as a metabolic stress defense. *JCI Insight*. 2019;4(4):e124079, 124079.

6. Cotter DG, et al. Ketogenesis prevents diet-induced fatty liver injury and hyperglycemia. *J Clin Invest*. 2014;124(12):5175–5190.

7. d'Avignon DA, et al. Hepatic ketogenic insufficiency reprograms hepatic glycogen metabolism and the lipidome. *JCI Insight*. 2018;3(12):e99762, 99762.

8. Wang M, Fang H, Han X. Shotgun lipidomics analysis of 4-hydroxyalkenal species directly from lipid extracts after one-step in situ derivatization. *Anal Chem*. 2012;84(10):4580–4586.

9. Puchalska P, et al. Determination of ketone bodies in biological samples via rapid UPLC-MS/MS. *Talanta*. 2021;225:122048.

10. Ayala JE, et al. Hyperinsulinemic-euglycemic clamps in conscious, unrestrained mice. *J Vis Exp JoVE*. 2011;(57):3188.

11. Rome FI, Hughey CC. Disrupted liver oxidative metabolism in glycine N-methyltransferase-deficient mice is mitigated by dietary methionine restriction. *Mol Metab*. 2022;58:101452.

12. Rome FI, et al. Loss of hepatic phosphoenolpyruvate carboxykinase 1 dysregulates metabolic responses to acute exercise but enhances adaptations to exercise training in mice. *Am J Physiol Endocrinol Metab*. 2023;324(1):E9–E23.

13. Hughey CC, et al. Loss of hepatic AMP-activated protein kinase impedes the rate of glycogenolysis but not gluconeogenic fluxes in exercising mice. *J Biol Chem*. 2017;292(49):20125–20140.

14. Hasenour CM, et al. Mass spectrometry-based microassay of (2)H and (13)C plasma glucose labeling to quantify liver metabolic fluxes in vivo. *Am J Physiol Endocrinol Metab*. 2015;309(2):E191-203.

15. Antoniewicz MR, Kelleher JK, Stephanopoulos G. Measuring deuterium enrichment of glucose hydrogen atoms by gas chromatography/mass spectrometry. *Anal Chem*. 2011;83(8):3211–3216.

16. Young JD. INCA: a computational platform for isotopically non-stationary metabolic flux analysis. *Bioinformatics*. 2014;30(9):1333–1335.

17. Fu X, et al. Targeted Determination of Tissue Energy Status by LC-MS/MS. *Anal Chem*. 2019;91(9):5881–5887.

18. Tr K, et al. Pyruvate-supported flux through medium-chain ketothiolase promotes mitochondrial lipid tolerance in cardiac and skeletal muscles. *Cell Metab*. 2023;35(6). https://doi.org/10.1016/j.cmet.2023.03.016.

19. Li Q, et al. Novel approach in LC-MS/MS using MRM to generate a full profile of acyl-CoAs: discovery of acyl-dephospho-CoAs. *J Lipid Res*. 2014;55(3):592–602.

20. Zhang G-F, et al. Catabolism of 4-hydroxyacids and 4-hydroxynonenal via 4-hydroxy-4-phosphoacyl-CoAs. *J Biol Chem*. 2009;284(48):33521–33534.

21. Williams AS, et al. Ketone flux through BDH1 supports metabolic remodeling of skeletal and cardiac muscles in response to intermittent time-restricted feeding. *Cell Metab*. 2024;36(2):422-437.e8.

22. Williams AS, et al. Disruption of Acetyl-Lysine Turnover in Muscle Mitochondria Promotes Insulin Resistance and Redox Stress without Overt Respiratory Dysfunction. *Cell Metab*. 2020;31(1):131-147.e11.

Supplemental Figure Legends

Supplemental Figure 1. Stable isotope delivery protocol for in vivo flux modeling in bariatric MASLD-MASH patients. For quantification of absolute flux through hepatic intermediary metabolic pathways subjects orally ingest three 250 mL doses of 70% heavy water (^2H_2O, D_2O) starting the night before flux measurements, then orally ingested two 300 mg doses of [U-$^{13}C_3$]propionate the morning of experiments. [3,4-$^{13}C_2$]glucose and D-[U-$^{13}C_4$]βOHB were then infused using a primed 2h continuous infusion with sample collection during the final 10 minutes after metabolic and isotopic steady state had been reached.

Supplemental Figure 2. BMI and insulin sensitivity in MASLD-MASH. Correlation of NAS score with **(A)** BMI, **(B)** acute insulin response to glucose (AIRg), and **(C)** the homeostatic model assessment for insulin resistance (HOMA-IR). Pearson correlations coefficients (r) are given on each graph along with a line of best fit and 95% confidence intervals calculated using linear regression. Correlations were accepted as significant if $p<0.05$ and are shown on each graph.

Supplemental Figure 3. Expected relationships among metabolic fluxes in humans illustrate robustness of the model. To demonstrate that metabolic fluxes followed logical biochemical patterns, Pearson correlation coefficients (r) were calculated to illustrate

connections within the metabolic network. **(A)** Correlation matrix for the eight in vivo fluxes shown in heat map format, with the magnitude of correlation shown by color and r displayed in each box. As expected, **(B)** pyruvate cycling (V_{PK+ME}), total anaplerosis and cataplerosis (V_{PEPCK}) and TCA cycle turnover (V_{CS}) were all positively correlated, and **(C)** positively correlated with endogenous glucose production (V_{EGP}). **(D)** V_{PK+ME}, V_{PEPCK}, and V_{CS} were positively correlated with the absolute flux of phosphoenolpyruvate (PEP) to glucose (V_{PEP}), but **(E)** were negatively correlated with fractional sourcing of glucose production from glycerol. Total fat oxidation was **(F)** strongly correlated with total ketogenesis (V_{RaTKB}), but **(G)** was not correlated with TCA cycle turnover (V_{CS}). Pearson correlations coefficients (r) are shown on each group along with a line of best fit and 95% confidence intervals calculated using linear regression. Correlations were accepted as significant if p<0.05 and are shown on each graph, or in each box on the correlation matrix an indicated, * p<0.05, ** p<0.01, ***p<0.001.

Supplemental Figure 4. Ketogenesis correlates with liver injury in humans. Correlation of NAS with absolute rates of hepatic glucose sourcing fluxes including **(A)** glycogenolysis ($V_{Glycogen}$), gluconeogenesis (GNG) from glycerol ($V_{Glycerol}$), and GNG from phosphoenolpyruvate (PEP) (V_{PEP}). **(B)** Correlation of NAS with absolute rates of pyruvate cycling (V_{PK+ME}) and total anaplerosis and cataplerosis (V_{PEPCK}). **(C)** Correlation of endogenous AcAc production rate (V_{RaAcAc}), total ketone body production rate (V_{RaTKB}), and total serum [^{12}C]ketone body concentrations with NAS score. V_{RaAcAc} was calculated as the product of $V_{Ra\beta OHB}$ and the ratio of total AcAc : total βOHB. V_{RaTKB} was calculated as the sum of V_{RaAcAc} + $V_{Ra\beta OHB}$. **(D)** Correlation of NAS score with the ratio of blood βOHB : AcAc. Pearson correlations coefficients (r) are given on each graph along with a line of best fit calculated using simple linear regression. Pearson correlations coefficients (r) are shown on each group along with a line of best fit and 95% confidence intervals calculated using linear regression. Correlations were accepted as significant if p<0.05 and are shown on each graph.

Supplementary Figure 5. Ketogenesis correlates with liver injury, independent of NEFAs. Correlation of $V_{Ra\beta OHB}$ with **(A)** PDFF**, (B)** AIRg, **(C)** serum AST and ALT. **(D)** Correlation of serum non-esterified fatty acids (NEFAs) concentrations with percent total body fat (left), percent android fat content (second from left), percent gynoid fat content (third from left), and the android/gynoid ratio (right). Pearson correlations coefficients (r) are given on each graph along with a line of best fit calculated using simple linear regression. Pearson correlations coefficients (r) are shown on each group along with a line of best fit and 95% confidence intervals calculated using linear regression. Correlations were accepted as significant if $p<0.05$ and are shown on each graph.

Supplementary Figure 6. Fat oxidation correlates with liver fibrosis in humans. The correlation of liver fibrosis score with **(A)** total fat oxidation, **(B)** RE production rate, **(C)** the ratio of anaplerosis relative to TCA cycle turnover (V_{PEPCK} / V_{CS}), and **(D)** the ratio of TCA cycle turnover relative to total glucose production (V_{CS} / V_{EGP}). Pearson correlations coefficients (r) are given on each graph along with a line of best fit calculated using simple linear regression. Pearson correlations coefficients (r) are shown on each group along with a line of best fit and 95% confidence intervals calculated using linear regression. Correlations were accepted as significant if $p<0.05$ and are shown on each graph.

Supplementary Figure 7. Loss of ketogenesis provokes a non-physiological rise in L-βOHB in HFCR-diet-fed mice. First the effect of feeding HFCR-diet on serum ketones was studied in WT mice. **(A)** AcAc, βOHB and total ketone bodies (TKBs) in chow or HFCR diet-fed WT mice after 4h fasting (n=6-8/group). **(B)** The ratio of βOHB:AcAc in chow or HFCR diet-fed WT mice after 4h fasting (n=6-8/group). **(C)** Absolute and **(D)** relative enantiomeric distribution of the serum βOHB pool in littermate WT control and HMGCS2-Liver-KO mice fed a HFCR diet (n=2-3/group). **(E)** Absolute TKB and **(F)** relative enantiomeric βOHB analysis of intrahepatic ketones from littermate WT control and HMGCS2-Liver-KO mice fed a HFCR diet and fasted for

4h (n=4-8/group). Data are expressed as mean ± standard deviation. Statistical differences were determined by Student's t-tests and accepted as significant if $p<0.05$. * $p<0.05$, ** $p<0.01$, *** $p<0.001$, as indicated. NS = not statistically significant. Data from chow-fed WT mice in panels A and B are the same data as those presented for chow-fed littermate control mice in **Figure 4C-D**. Data from HFCR diet-fed WT mice in panels A and B are the same data as those presented for HFCR diet-fed littermate control mice in **Figure 4E-F**.

Supplemental Figure 8. Loss of hepatocyte HMGCS2 disrupts hepatic energy metabolism in HFCR diet-fed mice. Metabolic fluxes were quantified in livers of female littermate control and HMGCS2-Liver-KO fasted mice fed HFCR diet for two days. **(A)** The ratio of total anaplerosis relative to TCA cycle turnover (V_{PCK} / V_{CS}), and the ratio of TCA cycle turnover relative to total glucose production (V_{CS} / V_{EGP}), in two-day HFCR diet-fed mice using in vivo $^{2}H/^{13}C$ isotope tracing (n=4-6/group). The pool size of various molecules that are central to hepatic energy metabolism were quantified in male and female HMGCS2-Liver-KO mice and their littermate controls fed HFCR diet for 1 week, including **(B)** acetyl-CoA, succinyl-CoA and propionyl-CoA, **(C)** individual energy adenylate nucleotides (ATP, ADP, AMP), and **(D)** total pool of all energy adenylate nucleotides calculated as [ATP] + [ADP] + [AMP] (n=4-8/group). **(E)** Liver energy charge calculated as ([ATP]+0.5[ADP]) / ([ATP]+[ADP]+[AMP]) (n=4-8/group). **(F)** Pool size of individual redox adenylate nucleotides (NAD^{+} and NADH), and **(G)** total pool of all redox adenylate nucleotides calculated as [NAD^{+}] + [NADH] (n=4-8/group). **(H)** Liver redox state calculated as [NAD^{+}] / [NADH] (n=4-8/group). Data are expressed as mean ± standard deviation. Statistical differences were determined by Student's t-tests and accepted as significant if $p<0.05$. * $p<0.05$, ** $p<0.01$, *** $p<0.001$, as indicated. NS = not statistically significant.

Supplemental Figure 9. Loss of hepatocyte BDH1 does not alter body weight, liver weight or fasting blood glucose in western diet-fed mice. Littermate control (WT) and BDH1-Liver-

KO male mice were fed a 42% kcal high-fat western diet (WD) for 16 weeks. **(A)** 18h fasted serum ketone body analysis, and **(B)** longitudinal random-fed weight gain (n=11-12/group). **(C)** 18h fasted liver weight and **(D)** blood glucose. **(E)** Total hepatic glucose production (V_{EGP}), **(F)** absolute and **(G)** relative sourcing of glucose in LCFA perfused livers from littermate WT control and BDH1-Liver-KO mice (n=10-11/group). Data are expressed as mean ± standard deviation. Statistical differences were determined by Student's t-tests and accepted as significant if $p<0.05$. * $p<0.05$, ** $p<0.01$, *** $p<0.001$, as indicated. NS = not statistically significant.

Supplemental Figure 10. Loss of hepatocyte BDH1 does not worsen liver injury in high-fat diet-fed mice. Littermate control (WT) and BDH1-Liver-KO male mice were fed a 60% high-fat diet (HFD) then **(A)** liver H and E (left) and picrosirius red (right) histological staining was used to assess liver health. **(B)** Liver fibrosis was quantified from the picrosirius staining (n=4-5/group). **(C)** Expression of liver genes related to fibrosis and inflammation (n=4-5/group). Littermate control (WT) and BDH1-Liver-KO male mice were fed choline-deficient, methionine-limited 62% kcal HFD then **(D)** liver H and E (left) and picrosirius red (right) histological staining was used to assess liver health. **(E)** Expression of liver genes related to fibrosis and inflammation (n=7-8/group). Scale bar in H&E images are 25 μm. Scale bar for picrosirius red images are 100 μm. Data are expressed as mean ± standard deviation. Statistical differences were determined by Student's t-tests and accepted as significant if $p<0.05$. * $p<0.05$, ** $p<0.01$, *** $p<0.001$, as indicated. NS = not statistically significant.

Supplementary Table 1: Inclusion and Exclusion Criteria for Human MASLD-MASH Subjects

Supplementary Table 2: RT-qPCR Primer Sequences. Sequences of primers for RT-qPCR analysis of mRNA transcript abundance, given in the 5' to 3' direction.

Supplemental Figure 1.

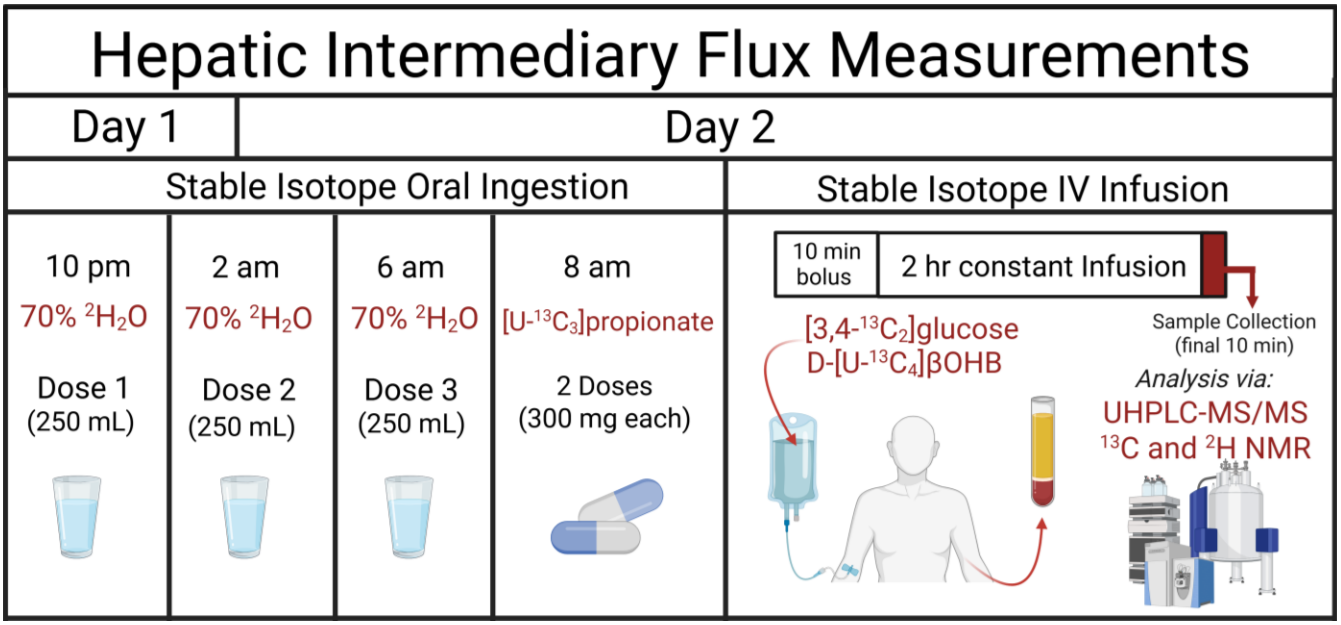

Supplemental Figure 2.

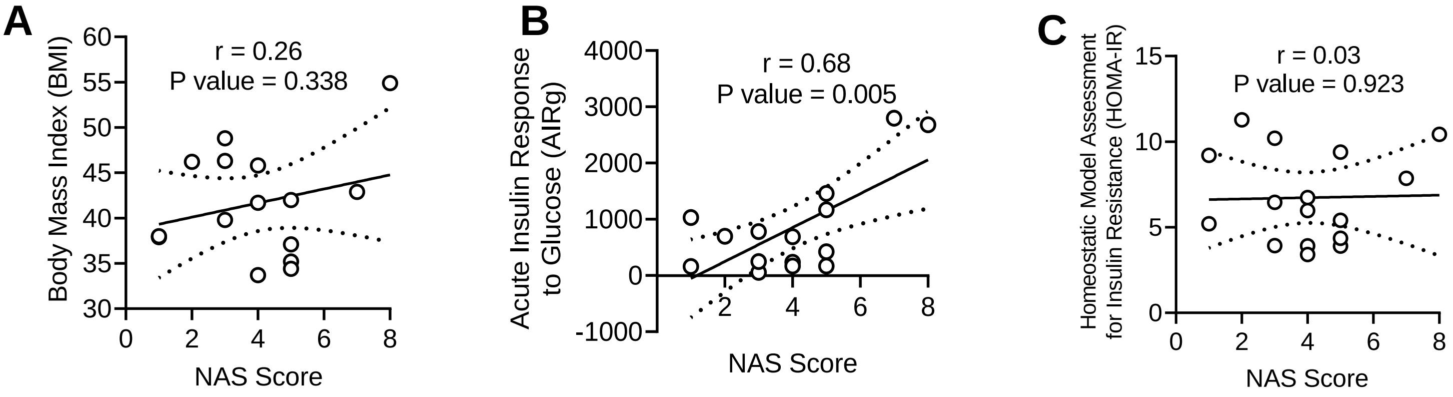

Supplemental Figure 3.

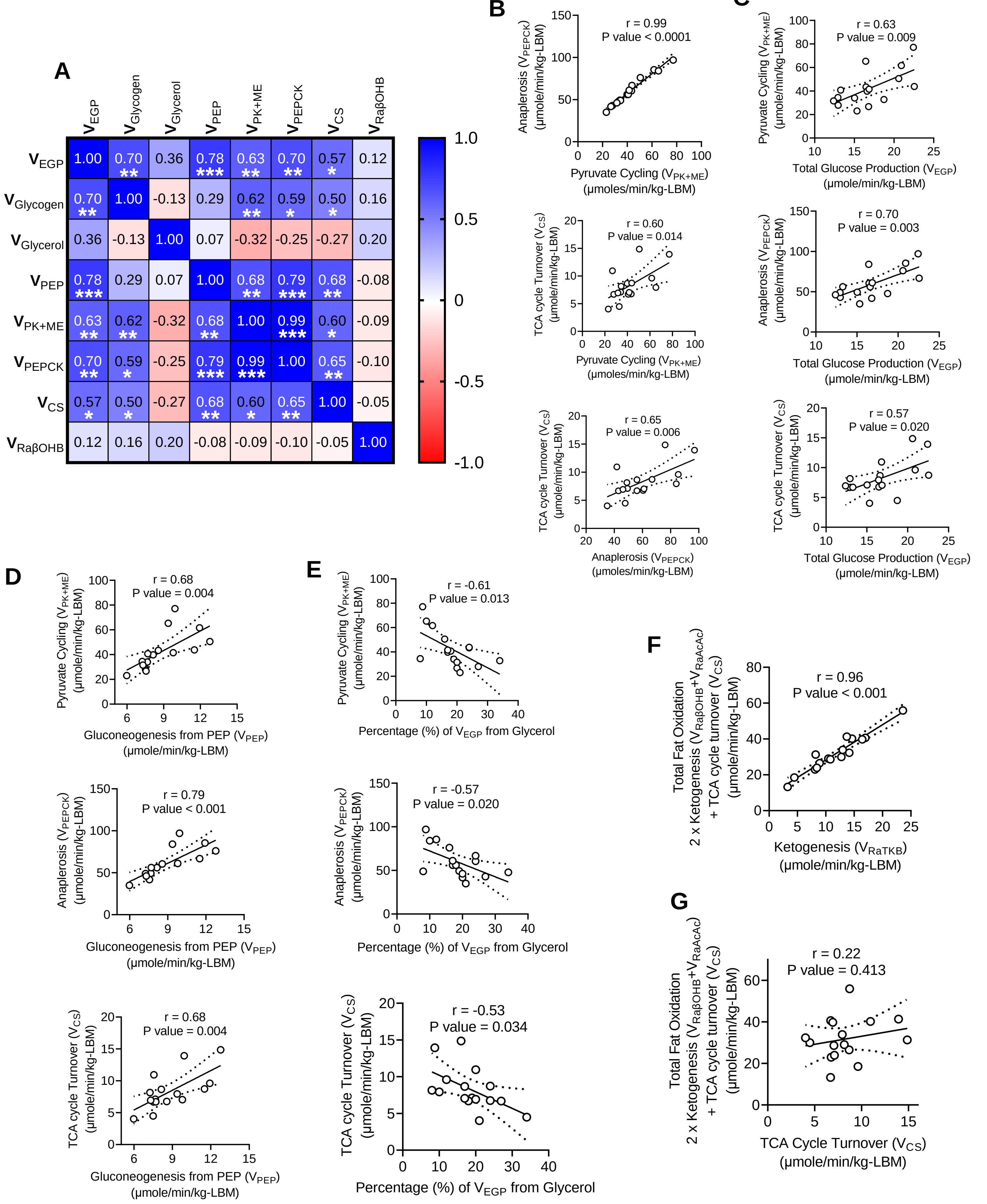

Supplemental Figure 4.

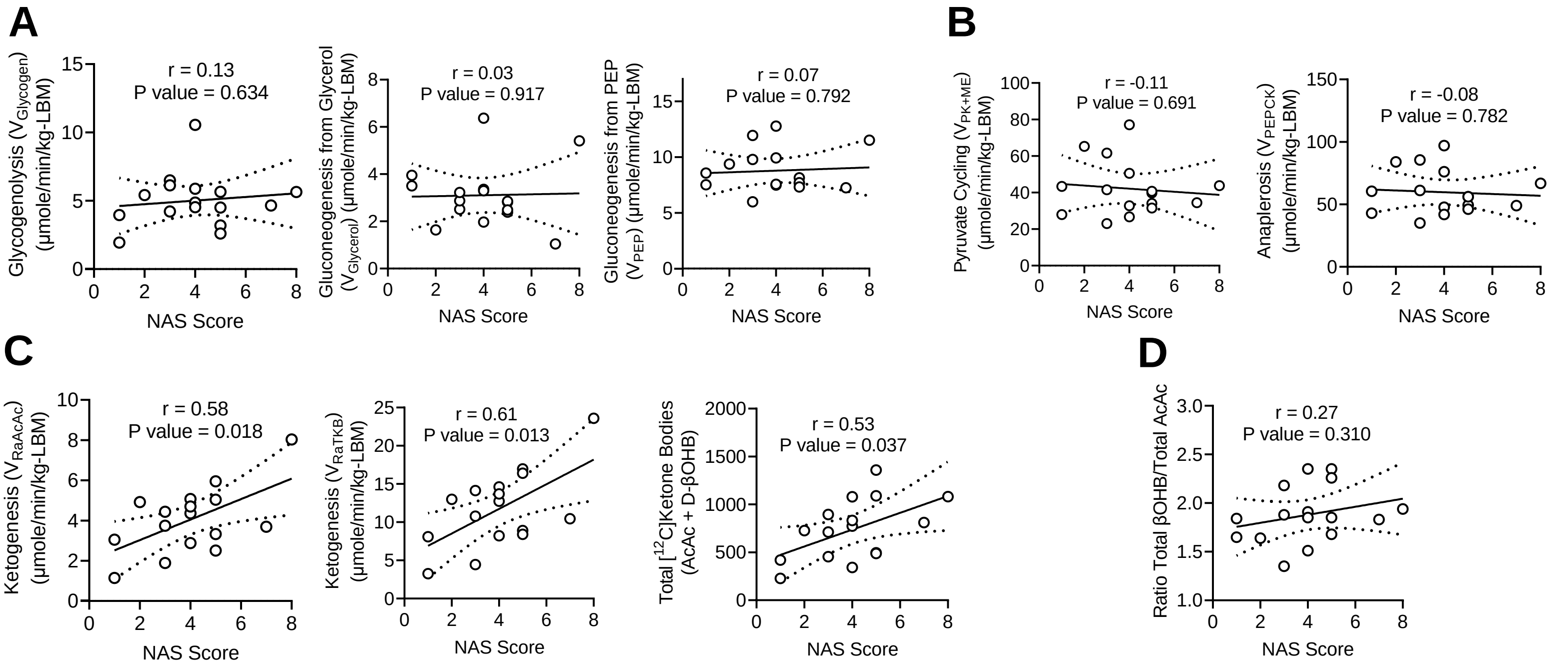

Supplemental Figure 5.

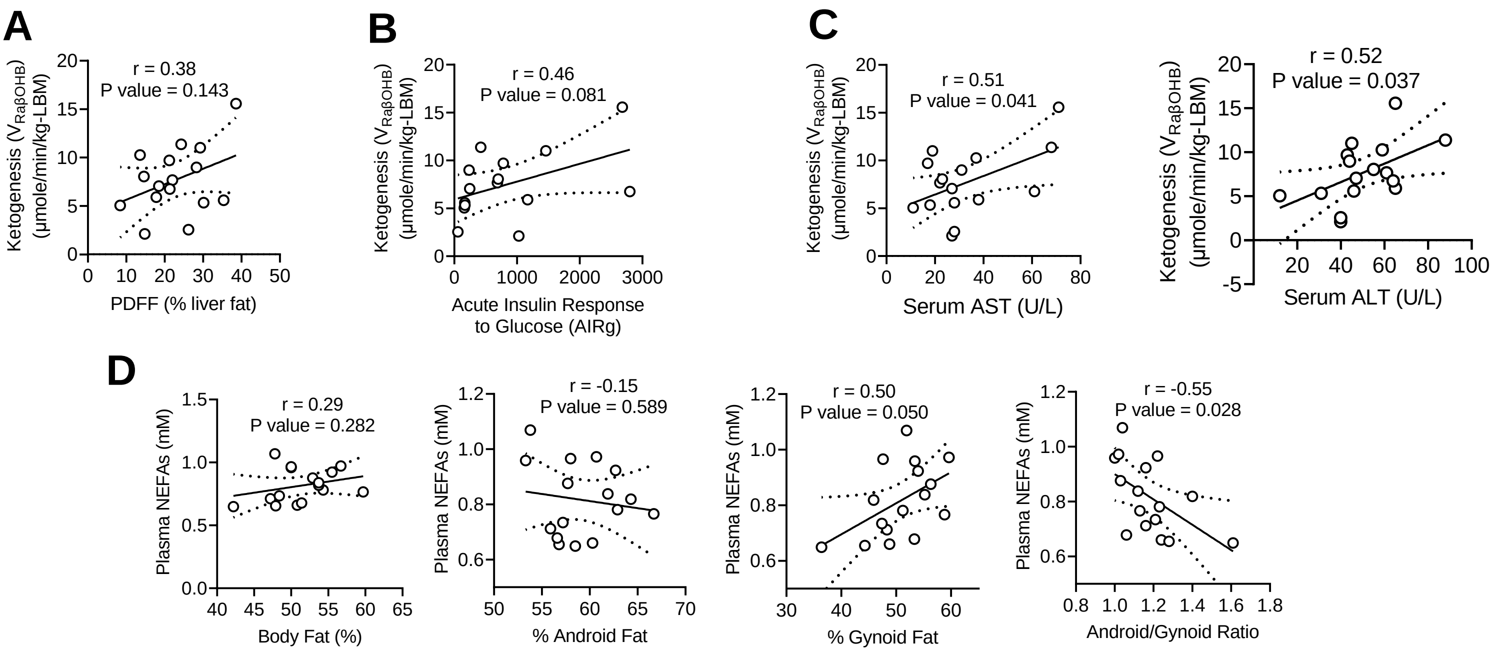

Supplemental Figure 6.

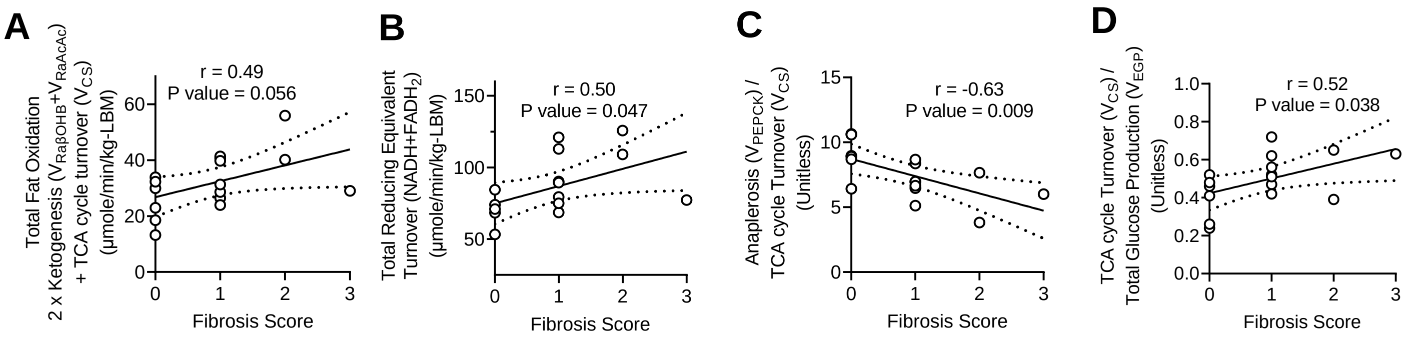

Supplemental Figure 7.

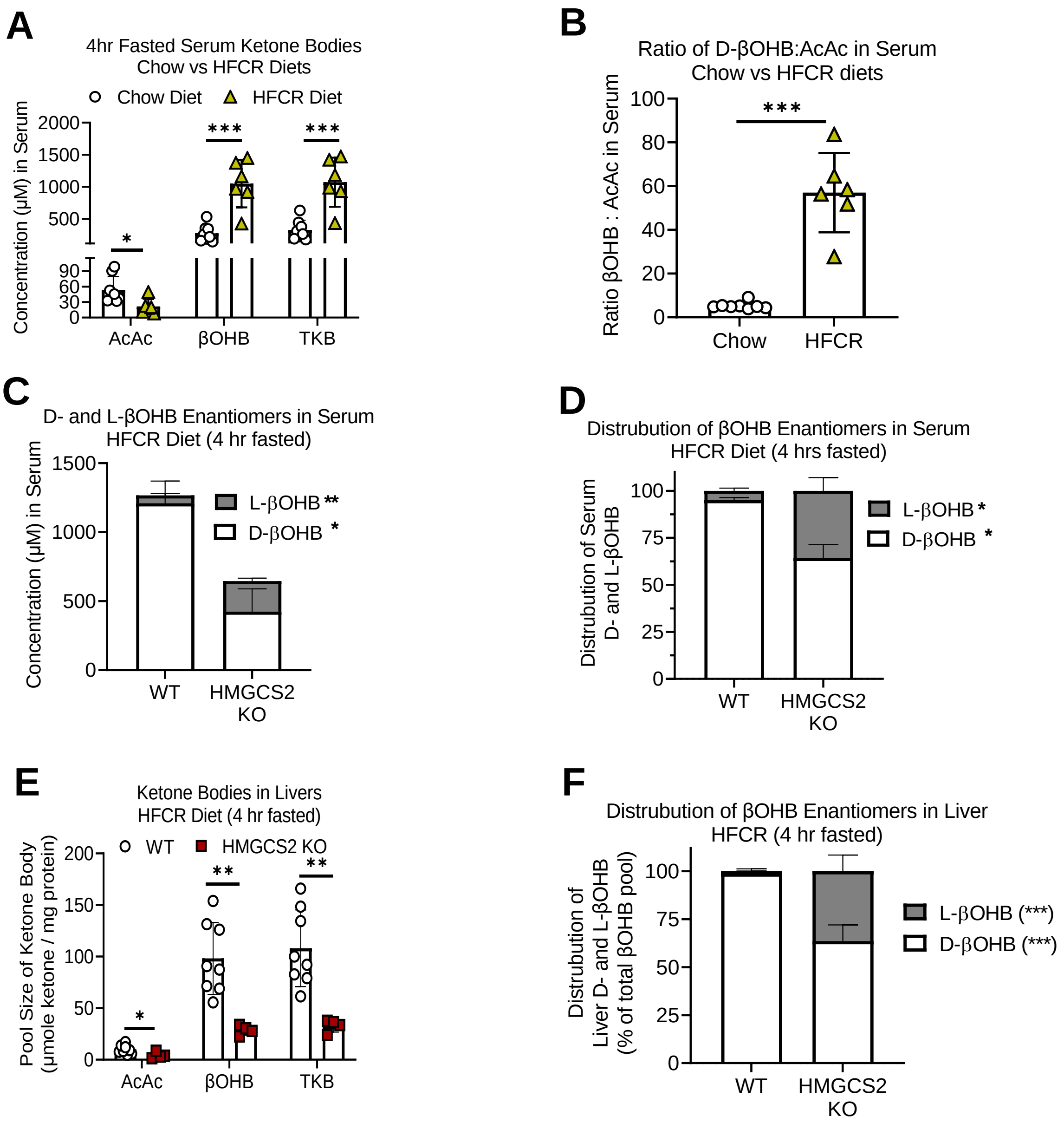

Supplemental Figure 8.

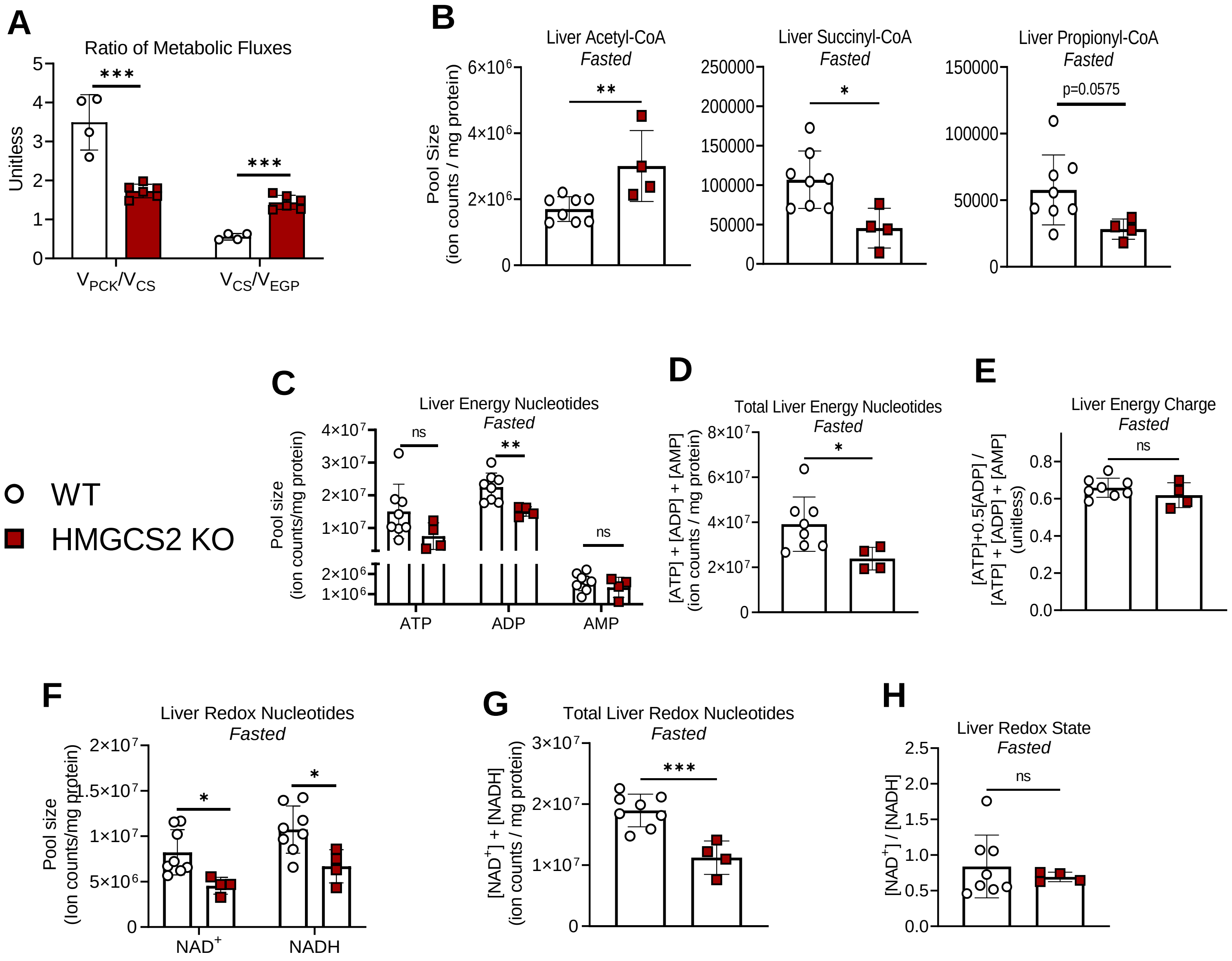

Supplemental Figure 9.

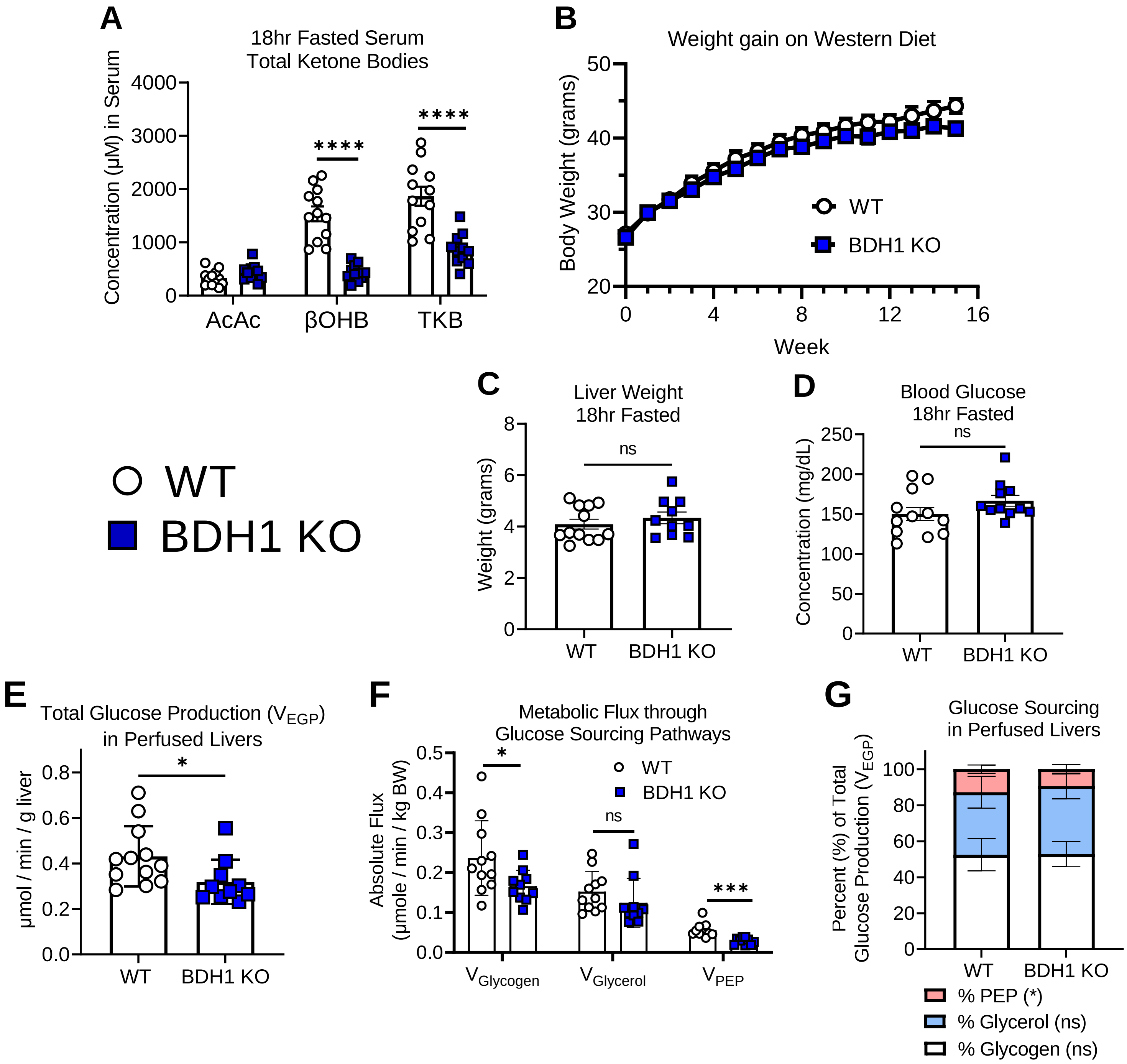

Supplemental Figure 10.

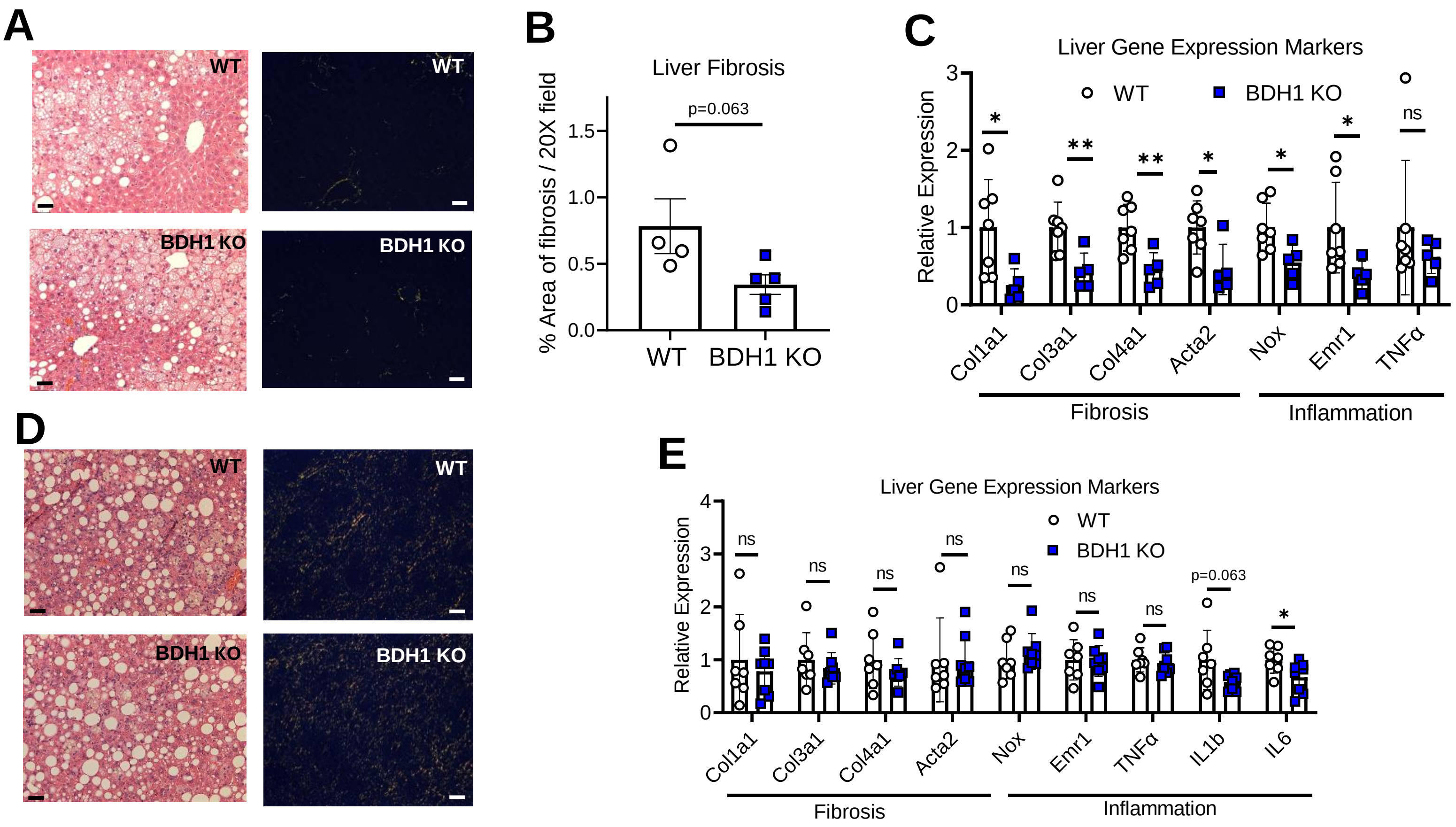

Supplement: Supplemental data [file jci-135-191021-s035.pdf]
